# Safety of SGLT2 inhibitors, DPP-4 inhibitors, and GLP-1 receptor agonists in US veterans with and without chronic kidney disease: a population-based study

**DOI:** 10.1016/j.lana.2024.100814

**Published:** 2024-06-18

**Authors:** Yoko Narasaki, Csaba P. Kovesdy, Amy S. You, Keiichi Sumida, Yamini Mallisetty, Satya Surbhi, Fridtjof Thomas, Alpesh N. Amin, Elani Streja, Kamyar Kalantar-Zadeh, Connie M. Rhee

**Affiliations:** a Department of Medicine, David Geffen School of Medicine at UCLA, Los Angeles, CA, USA; b Nephrology Section, Tibor Rubin Veterans Affairs Medical Center, Long Beach, CA, USA; c Division of Nephrology, Department of Medicine, University of Tennessee Health Science Center, Memphis, TN, USA; d Nephrology Section, Memphis Veterans Affairs Medical Center, Memphis, TN, USA; e Division of Hospital Medicine, Department of Medicine, University of California Irvine School of Medicine, Orange, CA, USA; f Division of Nephrology, Hypertension, and Kidney Transplantation, University of California Irvine, Orange, CA, USA; g The Lundquist Institute at Harbor-UCLA Medical Center, Torrance, CA, USA; h Nephrology Section, Veterans Affairs Greater Los Angeles Health Care System, Los Angeles, CA, USA

**Keywords:** SGLT2 inhibitors, DPP-4 inhibitors, GLP-1 receptor agonists, Safety, Adverse events

## Abstract

**Background:**

We examined the real-world comparative safety of sodium-glucose cotransporter-2 inhibitors (SGLT2i) vs. other newer anti-glycemic medications (dipeptidyl peptidase-4 inhibitors [DPP4i], glucagon-like peptide-1 receptor agonists [GLP1a]) in patients with and without chronic kidney disease (CKD).

**Methods:**

Among US Veterans with diabetes receiving care from the Veterans Affairs (VA) healthcare system over 2004–19, we identified incident users of SGLT2i vs. DPP4i vs. GLP1a monotherapy. In analyses stratified by CKD status, defined by estimated glomerular filtration rate and albuminuria, we examined associations of SGLT2i vs. DPP4i vs. GLP1a use with risk of infection-related (primary outcome) and genitourinary infection hospitalizations (secondary outcome) using multivariable Cox models.

**Findings:**

Among 92,269 patients who met eligibility criteria, 52% did not have CKD, whereas 48% had CKD. In the overall and non-CKD cohorts, compared to DPP4i use, SGLT2i use was associated with lower infection-related hospitalization risk (HRs [95% CIs] 0.74 [0.67–0.81] and 0.77 [0.67, 0.88], respectively), whereas GLP1a use demonstrated comparable risk. However, in the CKD cohort SGLT2i and GLP1a use were each associated with lower risk (HRs [95% CIs] 0.70 [0.61, 0.81] and 0.91 [0.84, 0.99], respectively). Propensity score-matched analyses showed similar findings in the non-CKD and CKD cohorts. In the overall, non-CKD, and CKD cohorts, SGLT2i use was associated with lower genitourinary infection hospitalization risk whereas GLP1a use showed comparable risk vs. DPP4i use.

**Interpretation:**

In a national cohort of Veterans with diabetes, compared with DPP4i use, SGLT2i use was associated with lower infection-related and genitourinary infection hospitalization risk.

**Funding:**

VA Health Services Research and Development, USA.

**Research in context**

**Evidence before this study**

While clinical trials show that SGLT2 inhibitors (SGLT2i) vs. placebo reduce risk of estimated glomerular filtration rate (eGFR) decline, end-stage kidney disease (ESKD), and renal- and cardiovascular-related mortality in chronic kidney disease (CKD) patients, there remain concerns regarding risk of potential complications based on non-CKD population data. We sought to examine the real-world safety of SGLT2i vs. other newer anti-glycemic medications (DPP-4 inhibitors [DPP4i], GLP1-agonists [GLP1a]) in patients with and without CKD. We searched PubMed and Google Scholar for studies examining newer anti-glycemic pharmacotherapies in patients with and without CKD which were published before June 2023 using the following search terms: (“sodium-glucose cotransporter-2 inhibitors” OR “glucagon-like peptide-1 receptor agonists” OR “dipeptidyl peptidase 4 inhibitors”) AND (“CKD” OR “kidney disease”).

**Added value of this study**

The findings of this study examining longitudinal health records from the national Veterans Affairs (VA) database add new knowledge regarding the real-world safety of newer anti-glycemic pharmacotherapies among US adults with and without CKD. Among 92,269 US Veterans with diabetes stratified by CKD status, we found that compared to incident DPP4i users, incident SGLT2i users had lower risk of infection-related hospitalizations in the overall, CKD, and non-CKD cohorts. While SGLT2i use was not associated with heightened risk of lower extremity amputation nor short-term acute kidney injury risk, there was higher risk of long-term acute kidney injury and diabetic ketoacidosis compared with DPP4i use.

**Implications of all the available evidence**

While a growing number of clinical trials have demonstrated robust cardiovascular and renal benefits of SGLT2i in both CKD and non-CKD patients, uptake of these medications in the real-world setting may in part be affected by the unclear safety of these medications in populations who are different than those included in these studies. Findings from this national study of US Veterans help inform the comparative safety profile of these anti-glycemic medications in the primary and secondary prevention of CKD.

## Introduction

To counteract the exceedingly high risk of cardiovascular and renal complications in patients with diabetes mellitus,^1^^,^^2^ there has been increasing emphasis of the use of novel anti-diabetic agents in this population.^3^, ^4^, ^5^, ^6^ This pharmacotherapeutic armamentarium includes sodium-glucose cotransporter-2 inhibitors (SGLT2i), a class of anti-diabetic medications that lower glucose through inhibition of the sodium-glucose cotransporter-2 channel (i.e., responsible for >90% of glucose reabsorption) in the proximal convoluted tubule of the kidney.^7^^,^^8^ In addition to its anti-glycemic effects, SGLT2i also exerts its cardio-renal benefits by restoring tubulo-glomerular feedback; reducing sodium reabsorption, glomerular hyperfiltration, and tubular workload/hypoxia; and decreasing renal inflammation and fibrosis.^3^^,^^7^, ^8^, ^9^

Based on a growing number of clinical trials demonstrating strong evidence for the efficacy of SGLT2i in reducing cardiovascular and renal complications in the general^10^, ^11^, ^12^ and CKD populations,^13^^,^^14^ this medication class has emerged as first-line therapy in diabetes.^15^^,^^16^ However, there remains ongoing debate regarding the safety profile of these medications, particularly in the context of underlying comorbidity burden.^17^^,^^18^ For example, while a higher frequency of lower extremity amputations^10^ and genitourinary infections^10^, ^11^, ^12^ were reported in SGLT2i trials in the general population, greater risk of these adverse events were not consistently observed in trials specific to the CKD population.^13^^,^^14^ Based on the physiologic effects of this class of medications, there is also concern about the potential risk of acute kidney injury (AKI)^17^ and euglycemic diabetic ketoacidosis (DKA),^8^^,^^11^ particularly in patients with CKD given their predisposition to these complications (i.e., via suppressed repair^19^ and impaired insulin secretion,^5^^,^^6^ respectively) independent of SGLT2i. Indeed, suboptimal uptake of SGLT2i^20^ may in part be due to limited understanding of the intermediate- and long-term risks of these complications in the real-world setting.

Hence, to address this knowledge gap, we sought to investigate the comparative safety of SGLT2i vs. other novel classes of anti-diabetic medications, namely glucagon-like peptide-1 receptor agonists (GLP1a) and dipeptidyl peptidase-4 inhibitors (DPP4i), among a large, contemporary cohort of US Veterans with detailed patient-level socio-demographic, comorbidity, medication, laboratory, and clinical data.^21^ Using this well-characterized cohort, we examined the occurrence of specific adverse events, including infection-related and genitourinary infection hospitalizations (primary and secondary outcomes, respectively), as well as AKI, lower extremity amputation, and DKA in sensitivity analyses, among incident/new users of SGLT2i in comparison to those who newly-initiated GLP1a and DPP4i, over an intermediate-to-long-term follow up period.

## Methods

### Source population

We conducted a historical cohort study using longitudinal data from the “*Therapeutic Interventions to Assess Outcomes and Disparities in Chronic Kidney Disease among Veterans (TRI-CKD)*” study, a retrospective cohort examining outcomes associated with various therapeutic interventions among US Veterans with, or at-risk for CKD.^21^, ^22^, ^23^, ^24^ Our source population consisted of 3,355,379 Veterans receiving care from the Veterans Affairs (VA) healthcare system over the period of October 1, 2004 through September 30, 2019. Patients were included in this study provided that they (1) were 18–90 years old at the time of study entry (i.e., defined as the first date of prescription of an SGLT2i, GLP1a, or DPP4i), (2) had a history of type 1 or 2 diabetes, (3) had one or more preceding estimated glomerular filtration rate (eGFR) and albuminuria measurements within 90-days of study entry (i.e., most proximate measurement designated as the baseline level), (4) were prescribed either an SGLT2i, GLP1a, or DPP4i, and (5) had at least one-year of prescription data prior to the first prescription of SGLT2i, GLP1a, or DPP4i (Supplementary Figs. S1–S4). Patients were excluded if they (6) were prescribed an SGLT2i, GLP1a, or DPP4i prior to study entry (i.e., in order to establish incident/new users of these medication classes), or (7) were prescribed more than one class of these medications (i.e., combined users of SGLT2i, GLP1a, or DPP4i). Criterion #6 was to address the possibility that patients receiving one of these medication classes at study entry were healthier than those who stopped or died using these medications prior to study entry due to adverse effects, which may bias results towards a protective effect; additionally, in contrast to a prevalent user design, the incident/new user design allows for appropriate temporal ordering of the exposure, outcome, and confounders; avoids adjustment for covariates affected by treatment; and also minimizes bias from reverse causation.^25^^,^^26^ The study was approved by the Institutional Review Board of the University of California Irvine Medical Center, VA Long Beach Healthcare System, and Memphis VA Medical Center.

### Exposure ascertainment

Our main objective was to compare the safety of SGLT2i vs. other newer anti-glycemic medications (GLP1a, DPP4i) in patients with and without underlying CKD. We examined incident/new users of SGLT2i vs. GLP1a vs. or DPP4i monotherapy. We collected information about prescribed medications from the Decision Support System National Data Extracts’ pharmacy files, as well as from Medicare Part D files for those eligible for such coverage.^27^

Given that these medications may have a differential impact according to underlying kidney function, we examined incident users of SGLT2i vs. GLP1a vs. DPP4i in the overall cohort, as well as among those stratified according to CKD status, namely (1) the “Non-CKD Cohort,” defined as patients with a baseline eGFR ≥ 60 ml/min/1.73 m^2^ and baseline urine-to-albumin creatinine ratio < 30 mg/g on or before study entry, and (2) the “CKD Cohort,” defined as patients with a baseline eGFR < 60 ml/min/1.73 m^2^ (including those with an eGFR < 30 ml/min/1.73 m^2^) or baseline albuminuria ≥30 mg/g on or before study entry. To investigate the safety of SGLT2i, GLP1a, vs. DPP4i across more granular stages of CKD, we further divided our CKD cohort into three subcategories according to their eGFR levels (stages 1–2, 3, vs. 4–5 CKD defined as eGFR levels ≥60, 30–<60, and <30 ml/min/1.73 m^2^, respectively) and conducted subgroup analyses for each outcome of interest.

### Outcome ascertainment

Our primary outcome of interest was (1) time to the first infection-related hospitalization following study entry, defined as the first hospitalization due to any infectious disease from categories 1 to 9 of the Agency for Healthcare Research and Quality (AHRQ) Clinical Classification System (CCS), namely gastrointestinal/liver, pulmonary, central nervous system, ophthalmic, human immunodeficiency virus, genitourinary, and rheumatologic, sepsis, and dermatologic etiologies.^28^ In secondary analyses, we examined (2) time to the first genitourinary infection hospitalization, and in sensitivity analyses we also evaluated the (3) frequency of any infection-related hospitalizations or genitourinary infection hospitalizations.

In sensitivity analyses, we also examined outcomes evaluated in clinical trials and observational studies of SGLT2i, including (4) time to the first AKI event following study entry, defined as an absolute increase in serum creatinine level of ≥0.3 mg/dl or a ≥50% increase in serum creatinine level from the baseline serum creatinine^29^ (i.e., defined as the most proximate creatinine on the date of or within one-year of study entry) within 48-hours of study entry based on time intervals of AKI ascertainment defined by the AKI Network criteria^30^ and previous epidemiologic studies of AKI^29^; in sensitivity analyses, we also explored time to the first AKI event within seven-, 30-, 60-, and 90-days after study entry. Additional outcomes included (5) time to the first lower extremity amputation event following study entry ascertained by procedure/diagnostic codes (Supplementary Table S1), and (6) time to the first DKA event following study entry ascertained by diagnostic codes (Supplementary Table S1).

For time-to-event analyses, follow-up began the day after study entry (i.e., defined as the first SGLT2i vs. GLP1a vs. DPP4i prescription date) and continued until occurrence of the outcome of interest, censoring event (i.e., death), or end of the study period (September 26th, 2019), whichever occurred first. Information on the outcomes of interest, censoring events, and the associated dates were obtained from the VA database, as well as United States Renal Data System data and Center for Medicare and Medicaid Services that were linked to the national VA database. For analyses examining frequency of infection-related hospitalizations or genitourinary infection hospitalizations, follow-up began the day after study entry and continued until occurrence of the outcome of interest, censoring event (i.e., death), or end of the study period (September 30th, 2019), whichever occurred first.

### Socio-demographic, comorbidity, and other treatment related information

Data from the VA and United States Renal Data System patient and medical evidence files were used to determine patients’ socio-demographic information (e.g., age, sex, race, ethnicity). Information regarding comorbidities were extracted from the VA Inpatient and Outpatient Medical Statistical Analysis System (SAS) datasets and Center for Medicare and Medicaid Services datasets using International Classification of Diseases, Ninth and Tenth Revision, Clinical Modification (ICD-9/10) diagnostic and procedure codes and Current Procedural Terminology codes.^31^ Charlson Comorbidity Index scores were estimated using the Deyo modification for administrative datasets without including kidney disease.^32^ Body mass index (BMI) data were obtained from the VA Vital Status file. Laboratory data except serum creatinine were obtained from the VA Decision Support System-National Data Extracts Laboratory Results.^33^ VA Corporate DataWarehouse LabChem data files were used to extract serum creatinine data.^34^ Using serum creatinine and demographic data, eGFR was calculated using the CKD Epidemiology Collaboration equation.^35^

### Statistical analyses

Baseline characteristics across exposure groups were compared as mean (SD) or median (IQR) values as dictated by data type. We first estimated the association between new users of SGLT2i vs. GLP1a vs. DPP4i and time-to-event outcomes (i.e., first overall or cause-specific infection-related hospitalization, AKI, amputation, and DKA event) using Cox proportional hazard models. We then examined the relationship between incident use of SGLT2i vs. GLP1a vs. DPP4i and frequency of infection-related hospitalizations (i.e., all-cause and genitourinary infection) defined by incident rate ratios (IRRs) using Poisson regression models. Both Cox and Poisson regression analyses were conducted using four incremental levels of covariate adjustment:

(1) *Unadjusted analyses* (Model 1): No adjustment for covariates;
(2) *Case-mix adjusted analyses* (Model 2): Adjusted for age, sex, race, ethnicity, myocardial infarction, congestive heart failure, cardiovascular disease, and Charlson Comorbidity Index;
(3) *Expanded case-mix adjusted analyses* (Model 3): Adjusted for case-mix covariates, plus eGFR, urine-to-albumin creatinine ratio, serum albumin, BMI, and glycated hemoglobin (HbA1c) levels;
(4) *Expanded case-mix + other anti-diabetes medications adjusted analyses* (Model 4): Adjusted for expanded case-mix covariates, plus insulin use vs. non-use and metformin use vs. non-use.

There were no missing values for age, sex, race, ethnicity, comorbidities (i.e., myocardial infarction, congestive heart failure, cardiovascular disease), eGFR, urine-to-albumin creatinine ratio, and use of anti-diabetes medications. The remaining covariates had ≤10% missing data, which included Charlson Comorbidity Index score (<1%), albumin (10%), BMI (1%), and HbA1c (2%). For the aforementioned covariates, missing data were addressed using multiple imputation with 12 imputed datasets, where multivariate normal distribution was assumed and variables without missing data in Model 4 were used as predictors of missing values. Proportional hazards assumptions were checked graphically using log–log plots. We also accounted for differences in baseline characteristics using 1:1 propensity score (PS) matching with a 0.2 matching caliper width. PSs were calculated using logistic regression models estimating the likelihood of anti-glycemic medication use by including covariates from the expanded case-mix + other anti-diabetes medications model. Statistical analyses were performed using SAS Enterprise Guide version 8.2 (SAS Institute Inc., Cary, NC, USA), Stata version 17.0 (Stata Corp., College Station, TX) and SigmaPlot version 13 (Systat Software, San Jose, CA).

### Role of the funding source

The funders had no role in study design, data collection, data analysis, interpretation, or writing of the report.

## Results

### Study population

In primary analyses examining the association between SGLT2i vs. GLP1a vs. DPP4i use and time to the first infection-related hospitalization, 92,269 patients met eligibility criteria (Supplementary Fig. S1). The mean (SD) age of the cohort was 68 (9) years, among whom 96% were male, 76% were White, and 8% were Hispanic. In the overall cohort, 14% (N = 13,126), 68% (N = 62,243), and 18% (N = 16,900) of patients were categorized as new users of SGLT2i, GLP1a, and DPP4i, respectively.

Baseline characteristics of the cohort stratified by CKD status are shown in Table 1. In the overall cohort, compared with new users of DPP4i, new users of GLP1a were more likely to have higher urine-to-albumin creatinine ratio levels, whereas new users of SGLT2i were more likely to have a history of myocardial infarction and higher eGFR levels. Compared to DPP4i users, those using SGLT2i and GLP1a were more likely to be of younger age; were less likely to have cardiovascular disease; had higher BMI and HbA1c levels; and were more likely to have insulin use. Across the three anti-diabetic medication exposure groups, there was a similar proportion of patients according to sex, race, and ethnicity, as well as similar levels of serum albumin. This pattern of baseline characteristics was similar in the CKD and non-CKD patients, except for the distribution of eGFR levels; whereas in the overall and CKD cohorts the baseline eGFR levels tended to be the highest among SGLT2i users, in the non-CKD cohort eGFR levels were similar across all three anti-diabetic medication exposure groups. Baseline characteristics of the patients in the AKI, amputation, and DKA subcohort analyses are shown in Supplementary Tables S2, S3, and S4, respectively. In the overall cohort, 11% of patients discontinued their anti-diabetic medication (i.e., proportion of patients who did not have second prescription after the index date or first prescription date).

**Table 1:** Baseline characteristics according to SGLT2i, DPP4i, and GLP1a use in the overall, CKD, and non-CKD cohorts.

	Overall				CKD				Non-CKD			
	Overall	Anti-DM Medication			Overall	Anti-DM Medication			Overall	Anti-DM Medication		
		SGLT2i	DPP4i	GLP1a		SGLT2i	DPP4i	GLP1a		SGLT2i	DPP4i	GLP1a
N (%) of participants	92,269 (100)	13,126 (14.2)	62,243 (67.5)	16,900 (18.3)	44,036 (100)	5583 (12.7)	29,382 (66.7)	9071 (20.6)	48,233 (100)	7543 (15.6)	32,861 (68.1)	7829 (16.2)
Age (years), mean (SD)	68 (9)	67 (8)	69 (10)	65 (9)	70 (9)	69 (8)	72 (9)	67 (8)	66 (9)	66 (8)	67 (9)	63 (9)
Male, N (%)	88,201 (96)	12,669 (97)	59,728 (96)	15,804 (94)	42,595 (97)	5434 (97)	28,523 (97)	8638 (95)	45,606 (95)	7235 (96)	31,205 (95)	7166 (92)
White race, N (%)	70,040 (76)	10,143 (77)	47,130 (76)	12,767 (76)	33,658 (76)	4313 (77)	22,404 (76)	6941 (77)	36,382 (75)	5830 (77)	24,726 (75)	5826 (74)
Black race, N (%)	16,683 (18)	2287 (17)	11,270 (18)	3126 (18)	7416 (17)	956 (17)	4913 (17)	1547 (17)	9267 (19)	1331 (18)	6357 (19)	1579 (20)
Other race, N (%)	1812 (2)	263 (2)	1242 (2)	307 (2)	911 (2)	128 (2)	606 (2)	177 (2)	901 (2)	135 (2)	636 (2)	130 (2)
Missing race, N (%)	0 (0)	0 (0)	0 (0)	0 (0)	0 (0)	0 (0)	0 (0)	0 (0)	0 (0)	0 (0)	0 (0)	0 (0)
Asian/Pacific Islander race, N (%)	2128 (2)	262 (2)	1463 (2)	403 (2)	1103 (3)	119 (2)	765 (3)	219 (2)	1025 (2)	143 (2)	698 (2)	184 (2)
Hispanic ethnicity, N (%)	7415 (8)	1192 (9)	4988 (8)	1235 (7)	3066 (7)	458 (8)	2029 (7)	579 (6)	4349 (9)	734 (10)	2959 (9)	656 (8)
eGFR (mL/min/1.73 m^2^), mean (SD)	72 (21)	76 (18)	71 (21)	71 (23)	59 (21)	67 (19)	58 (20)	58 (22)	83 (14)	83 (14)	83 (14)	85 (15)
UACR (mg/g), median (IQR)	60 (12,48,718)	61 (12,48,718)	51 (11,34,128)	78 (18,26,933)	151 (56,48,718)	183 (67,48,718)	120 (47,29,278)	236 (67,20,172)	8 (0,30)	8 (0,30)	8 (0,30)	9 (0,30)
Albumin (g/dl), mean (SD)	4.0 (0.4)	4.0 (0.4)	4.0 (0.4)	3.9 (0.4)	3.9 (0.4)	3.9 (0.4)	3.9 (0.4)	3.8 (0.4)	4.0 (0.4)	4.0 (0.4)	4.0 (0.4)	4.0 (0.4)
BMI (kg/m^2^), mean (SD)	32.8 (6.6)	33.4 (6.3)	31.6 (6.1)	36.6 (7.1)	32.7 (6.7)	33.4 (6.3)	31.4 (6.1)	36.6 (7.0)	32.8 (6.6)	33.3 (6.3)	31.7 (6.1)	36.7 (7.2)
HbA1c, N (%)	8.5 (1.6)	8.6 (1.4)	8.3 (1.6)	9.0 (1.6)	8.5 (1.6)	8.7 (1.4)	8.3 (1.5)	9.0 (1.5)	8.4 (1.6)	8.6 (1.4)	8.3 (1.7)	8.9 (1.6)
MI, N (%)	19,991 (22)	3391 (26)	13,095 (21)	3505 (21)	11,493 (26)	1612 (29)	7518 (26)	2363 (26)	8498 (18)	1779 (24)	5577 (17)	1142 (15)
CHF, N (%)	27,246 (30)	3674 (28)	18,532 (30)	5040 (30)	16,608 (38)	1875 (34)	11,267 (38)	3466 (38)	10,638 (22)	1799 (24)	7265 (22)	1574 (20)
CVD, N (%)	21,368 (23)	2785 (21)	15,409 (25)	3174 (19)	12,203 (28)	1368 (25)	8744 (30)	2091 (23)	9165 (19)	1417 (19)	6665 (20)	1083 (14)
Charlson Comorbidity Index, mean (SD)	5 (3)	5 (3)	5 (3)	5 (3)	6 (3)	5 (3)	6 (3)	6 (3)	4 (3)	4 (3)	5 (3)	4 (3)
Insulin, N (%)	66,916 (73)	10,081 (77)	41,254 (66)	15,581 (92)	33,794 (77)	4528 (81)	20,690 (70)	8576 (95)	33,122 (69)	5553 (74)	20,564 (63)	7005 (89)
Metformin, N (%)	88,693 (96)	12,797 (97)	59,482 (96)	16,414 (97)	41,684 (95)	5426 (97)	27,535 (94)	8723 (96)	47,009 (97)	7371 (98)	31,947 (97)	7691 (98)

Continuous variables are presented as means ± SDs or medians (IQRs) as dictated by data type and categorical variables are presented as column percentages.

CKD was defined as a baseline eGFR < 60 ml/min/1.73 m^2^ (including an eGFR < 30 ml/min/1.73 m^2^) or baseline albuminuria ≥30 mg/g on or before study entry.

CHF, Congestive heart failure; CKD, Chronic kidney disease; CVD, Cardiovascular disease; DM, diabetes mellitus; DPP4i, dipeptidyl peptidase-4 inhibitor; GLP1a, glucagon-like peptide-1 receptor agonist; MI, Myocardial infarction; SGLT2i, sodium-glucose cotransporter-2 inhibitor; UACR, Urine Albumin-to-Creatinine Ratio.

### SGLT2i vs. GLP1a vs. DPP4i and infection-related hospitalizations

In analyses of the overall cohort, patients contributed a total of 245,720 person-years of follow-up, during which time 10,140 infection-related hospitalizations of any cause occurred (crude rate: 10.99 events per 1000 person-years of follow up). Median (IQR) at-risk time was 1.9 (0.8, 3.8) years, and the proportion of death and end-stage kidney disease (ESKD) events in the cohort are shown in Supplementary Table S5. In the CKD cohort, patients contributed a total of 105,214 person-years of follow-up, during which time 5600 infection-related hospitalizations occurred (crude rate: 12.72 events per 1000 person-years of follow up), and median (IQR) at-risk time was 1.7 (0.7, 3.4) years. In the non-CKD cohort, patients contributed a total of 140,506 person-years of follow-up, during which time 4540 all-cause infection-related hospitalizations occurred (crude rate: 9.41 events per 1000 person-years of follow up), and median (IQR) at-risk time was 2.2 (0.9, 4.2) years.

**Fig. 1:**
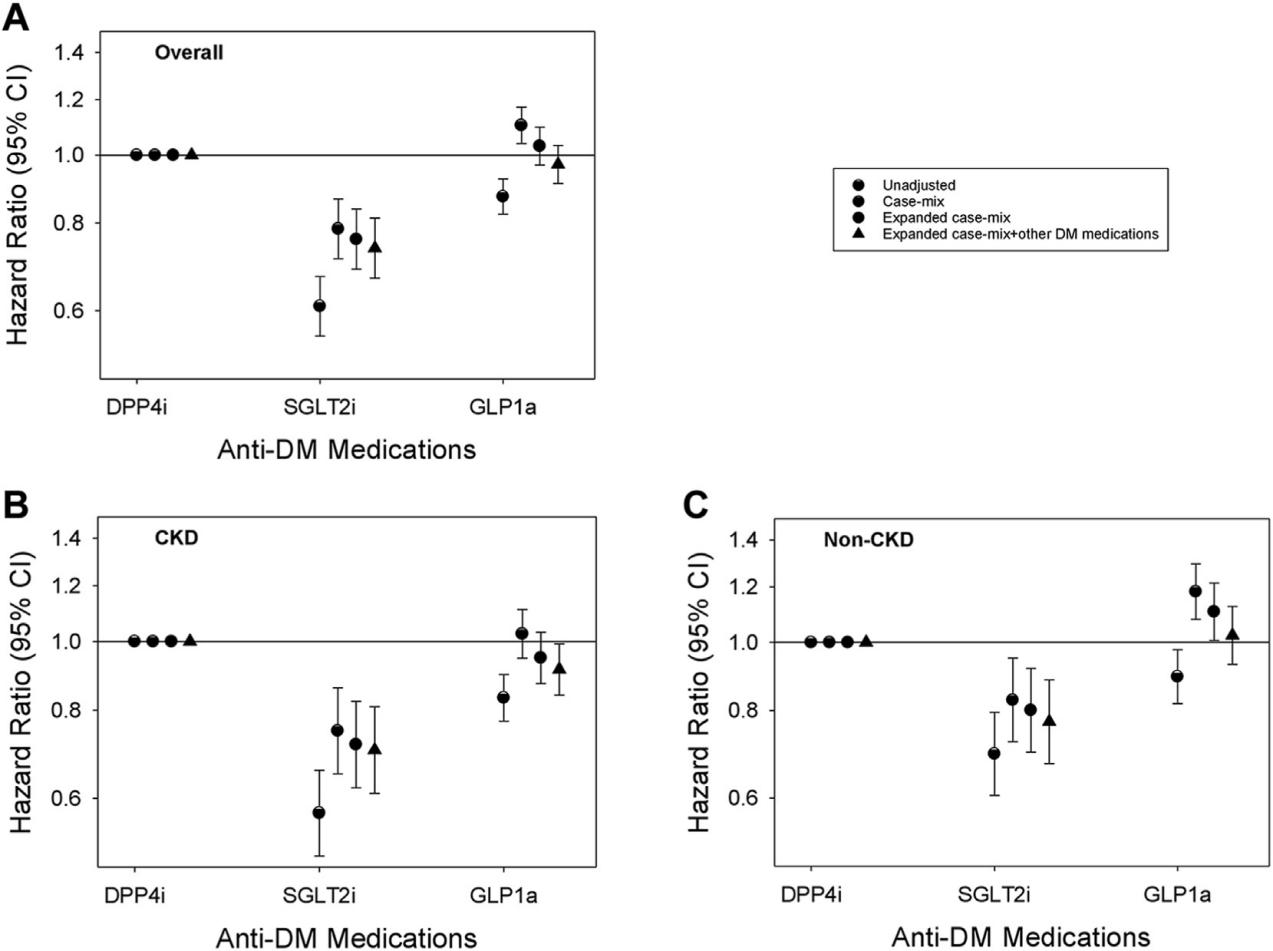
**Association of SGLT2i vs. GLP1a vs. DPP4i medication use with time to the first infection-related hospitalization in the overall cohort (Panel A; N = 92,269), CKD cohort (Panel B; N = 44,036), and non-CKD cohort (Panel C; N = 48,233).** Each plot shows hazard ratios and their 95% CIs estimated using Cox regression models incrementally adjusted with the following covariates: (1) Unadjusted analyses; (2) Case-mix analyses adjusted for age, sex, race, ethnicity, myocardial infarction, congestive heart failure, cardiovascular disease, and Charlson Comorbidity Index; (3) Expanded case-mix analyses adjusted for case-mix covariates, plus estimated glomerular filtration rate, urine albumin-to-creatinine ratio, serum albumin, body mass index, and glycated hemoglobin; and (4) Expanded case-mix + other anti-diabetes medications analyses adjusted for expanded case-mix covariates, plus insulin and metformin. Abbrev.: CKD, chronic kidney disease; DPP4i, dipeptidyl peptidase-4 inhibitor; GLP1a, glucagon-like peptide-1 receptor agonist; SGLT2i, sodium-glucose cotransporter-2 inhibitor.

In time-to-event analyses of the overall cohort, compared to DPP4i users, SGLT2i users had lower risk of infection-related hospitalizations (adjusted HR [aHR] [95% CI] 0.74 [0.67, 0.81]), whereas GLP1a users had similar risk (aHR [95% CI] 0.97 [0.91, 1.03]) in Cox models adjusted for expanded case-mix + laboratory + other anti-diabetic medication covariates (Fig. 1 and Supplementary Table S6). Similar findings were observed in the non-CKD cohort. However, in the CKD cohort, compared to DPP4i users, SGLT2i and GLP1a users both had lower risk of infection-related hospitalizations: aHRs (95% CIs) 0.70 (0.61, 0.81) and 0.91 (0.84, 0.99), respectively, in expanded case-mix + laboratory + other anti-diabetic medication Cox models (Fig. 1 and Supplementary Table S6). In PS-matched analyses, we observed a similar pattern of findings in the non-CKD and CKD cohorts (Supplementary Table S7). In analyses examining more granular stages of CKD, SGLT2i users had lower risk of infection-related hospitalizations compared to DPP4i users among patients with stages 1–2 and 3 CKD (Supplementary Table S8). Due to the small number of SGLT2i users with stages 4–5 CKD (N = 19), we did not perform analysis of this subgroup. When comparing GLP1a vs. DPP4i users, GLP1a had similar risk of infection-related hospitalizations among those with stages 1–2 and 3 CKD but lower risk among those with stages 4–5 CKD.

**Fig. 2:**
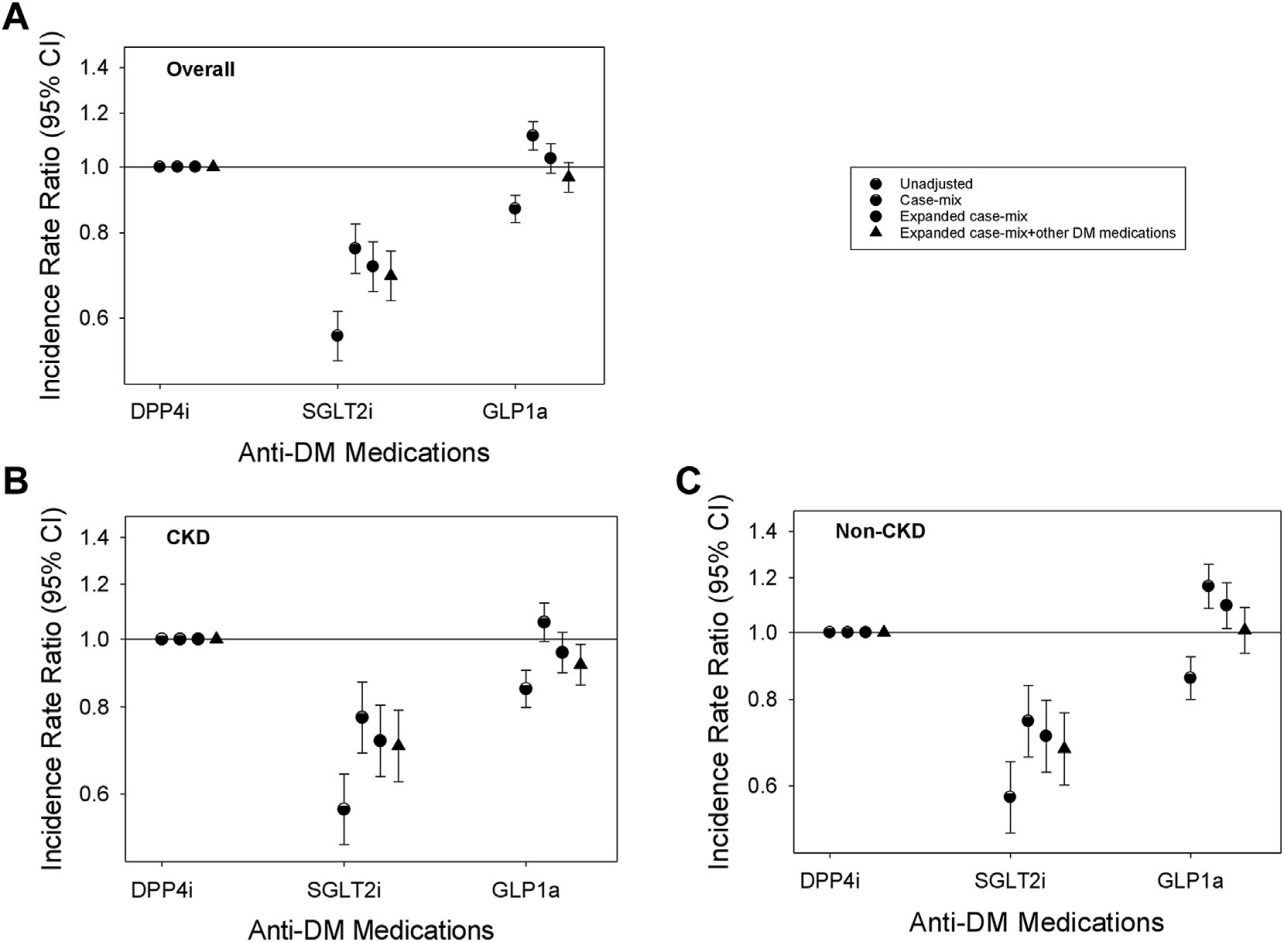
**Association of SGLT2i vs. GLP1a vs. DPP4i medication use with frequency of infection-related hospitalizations in the overall cohort (Panel A; N = 92,269), CKD cohort (Panel B; N = 44,036), and non-CKD cohort (Panel C; N = 48,233).** Each plot shows hazard ratios and their 95% CIs estimated using Cox regression models incrementally adjusted with the following covariates: (1) Unadjusted analyses; (2) Case-mix analyses adjusted for age, sex, race, ethnicity, myocardial infarction, congestive heart failure, cardiovascular disease, and Charlson Comorbidity Index; (3) Expanded case-mix analyses adjusted for case-mix covariates, plus estimated glomerular filtration rate, urine albumin-to-creatinine ratio, serum albumin, body mass index, and glycated hemoglobin; and (4) Expanded case-mix + other anti-diabetes medications analyses adjusted for expanded case-mix covariates, plus insulin and metformin. Abbrev.: CKD, chronic kidney disease; DPP4i, dipeptidyl peptidase-4 inhibitor; GLP1a, glucagon-like peptide-1 receptor agonist; SGLT2i, sodium-glucose cotransporter-2 inhibitor.

In sensitivity analyses examining the frequency of infection-related hospitalizations in the overall cohort, SGLT2i use was associated with a lower IRR of infection-related hospitalizations (ref: DPP4i use) (adjusted IRR [aIRR] [95% CI] 0.69 [0.64, 0.75]), whereas GLP1a was associated with a similar risk (aIRR [95% CI] 0.97 [0.92, 1.02]) in expanded case-mix + laboratory + other anti-diabetic medication analyses (Fig. 2 and Supplementary Table S9). A similar pattern of findings was observed in the non-CKD cohort. In the CKD cohort, lower IRRs of infection-related hospitalizations were observed for both SGLT2i and GLP1a use (ref: DPP4i use): aIRRs (95% CIs) 0.70 (0.62, 0.79) and 0.92 (0.86, 0.98), respectively, in expanded case-mix + laboratory + other anti-diabetic medication analyses. In PS-matched analyses, we observed a similar pattern of findings in the non-CKD and CKD cohorts (Supplementary Table S7). In analyses examining more granular stages of CKD, SGLT2i users had lower IRRs of infection-related hospitalizations compared to DPP4i users among patients with stages 1–2 and 3 CKD (Supplementary Table S8). When comparing GLP1a vs. DPP4i users, GLP1a had similar IRRs of infection-related hospitalizations among those with stages 1–2 and 3 CKD but lower IRR among those with stages 4–5 CKD.

### SGLT2i vs. GLP1a vs. DPP4i and genitourinary infection hospitalizations

In secondary analyses examining time to cause-specific infection hospitalizations, compared to DPP4i users, SGLT2i users had lower risk of genitourinary infection hospitalizations in the overall (aHR [95% CI] 0.62 [0.46,0.83]), CKD (aHR [95% CI] 0.66 [0.44, 1.00]), and non-CKD cohorts (aHR [95% CI] 0.56 [0.36, 0.87]) in expanded case-mix + laboratory + other anti-diabetic medication Cox models (Supplementary Fig. S5 and Table S10). In PS-matched analyses, we observed a similar pattern of findings in the non-CKD and CKD cohorts, except SGLT2i vs. DPP4i use showed similar risk in the non-CKD cohort (Supplementary Table S7). In analyses examining more granular stages of CKD, SGLT2i users had lower risk of genitourinary infection hospitalizations compared to DPP4i users among patients with stages 1–2 CKD (Supplementary Table S8).

In sensitivity analyses examining the frequency of cause-specific infection-related hospitalizations in the overall cohort, SGLT2i use was associated with lower IRRs of genitourinary infection-related hospitalizations (ref: DPP4i use): aIRR (95% CI) 0.55 (0.41,0.72) in expanded case-mix + laboratory + other anti-diabetic medication Cox analyses (Supplementary Fig. S6 and Table S11). A similar pattern of findings was observed in the CKD and non-CKD cohorts. In PS-matched analyses, we observed a similar pattern of findings in the non-CKD and CKD cohorts (Supplementary Table S7). In analyses examining more granular stages of CKD, SGLT2i users had lower risk of genitourinary infection hospitalizations compared to DPP4i users among patients with stages 1–2 CKD (Supplementary Table S8).

### SGLT2i vs. GLP1a vs. DPP4i and risk of acute kidney injury

We also examined the association between SGLT2i, GLP1a, and DPP4i use and AKI risk within 48-hours following study entry (i.e., initial date of SGLT2i, GLP1a, or DPP4i prescription). In the overall, CKD, and non-CKD cohorts, there were 5715, 3062, and 2653 patients who had available serum creatinine measurements for these analyses (Supplementary Table S12). In the overall cohort, compared to DPP4i use, SGLT2i and GLP1a were not associated with higher risk of AKI within 48-hours of initial prescription in expanded case-mix + laboratory + other anti-diabetic medication Cox models. A similar pattern of findings was observed in the CKD and non-CKD cohorts. In PS-matched analyses, we observed a similar pattern of findings in the overall, non-CKD, and CKD cohorts (Supplementary Table S13). In analyses examining granular CKD subcategories, SGLT2i and GLP1a were not associated with higher risk of AKI within 48-hours of initial medication prescription among patients with stages 1–2, 3, and 4–5 CKD (Supplementary Table S14). The proportion of drug discontinuation according to AKI status within 48-hours of initial prescription are shown in Supplementary Table S15.

We also conducted sensitivity analyses examining AKI risk across varying time intervals following study entry. In the overall cohort, there were a total of 8232, 19,921, 33,763, and 48,188 patients who had available serum creatinine measurements for assessment of AKI events within seven-, 30, 60-, and 90-days of initial prescription (Supplementary Table S12). When examining AKI risk within seven-days of prescription, SGLT2i use was associated with higher risk of AKI in the overall and non-CKD cohorts (aHRs [95% CI] 1.49 [1.16, 1.92] and 1.75 [1.16, 2.64], respectively), but not in the CKD cohort (aHR [95% CI] 1.35 [0.97, 1.86]) (ref: DPP4i use). Yet when examining AKI-risk over longer time intervals, SGLT2i was associated with higher risk within 30-, 60- and 90-days of prescription vs. DPP4i use in the overall, CKD, and non-CKD cohorts. Compared with DPP4i use, GLP1a use was associated with higher risk of AKI within 30-, 60- and 90-days of prescription in the non-CKD cohort only.

### SGLT2i vs. GLP1a vs. DPP4i and risk of amputation

**Fig. 3:**
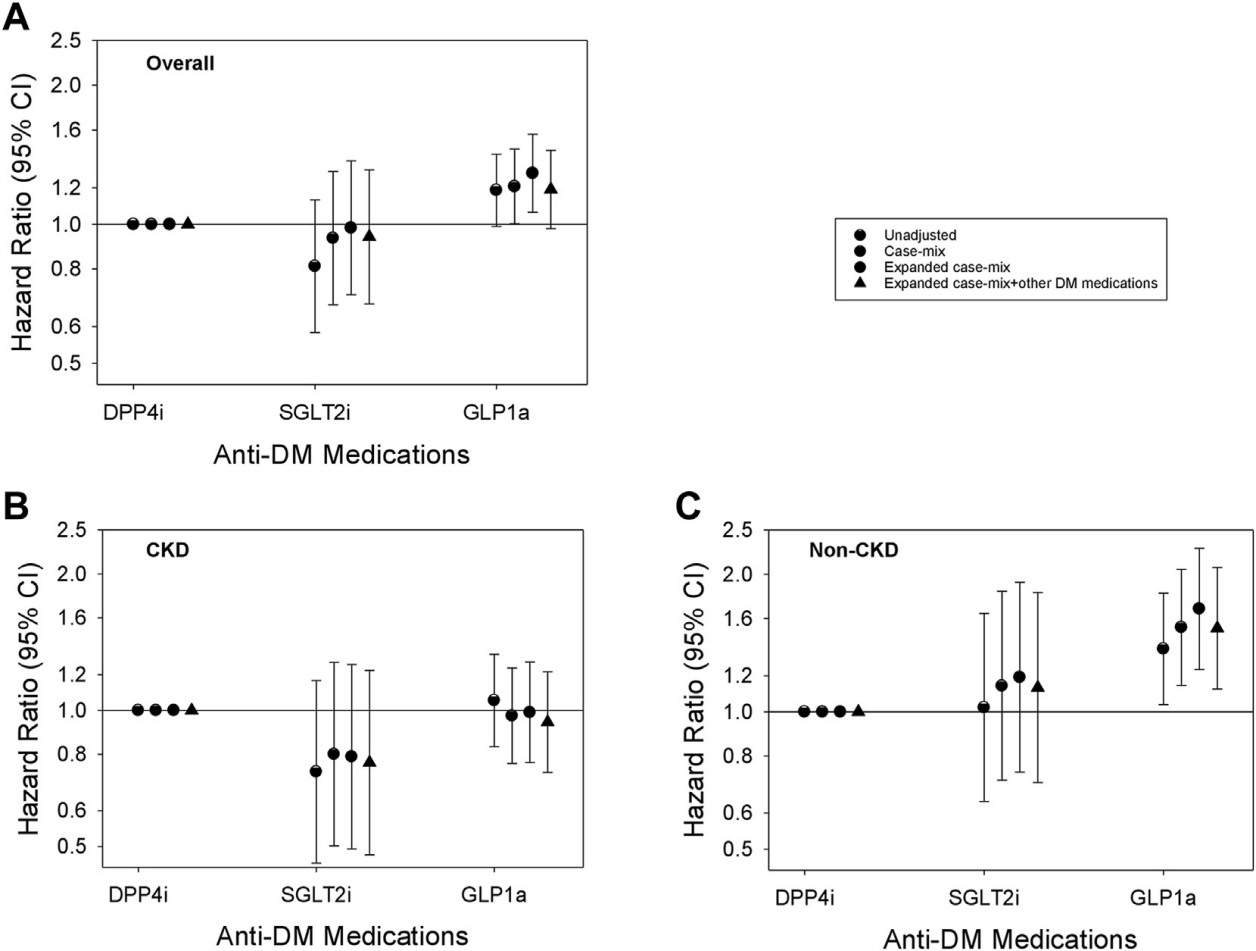
**Association of SGLT2i vs. GLP1a vs. DPP4i medication use with risk of lower extremity amputation in the overall cohort (Panel A; N = 88,281), CKD cohort (Panel B; N = 41,917), non-CKD cohort (Panel C; N = 46,364).** Each plot shows hazard ratios and their 95% CIs estimated using Cox regression models incrementally adjusted with the following covariates: (1) Unadjusted analyses; (2) Case-mix analyses adjusted for age, sex, race, ethnicity, myocardial infarction, congestive heart failure, cardiovascular disease, and Charlson Comorbidity Index; (3) Expanded case-mix analyses adjusted for case-mix covariates, plus estimated glomerular filtration rate, urine albumin-to-creatinine ratio, serum albumin, body mass index, and glycated hemoglobin; and (4) Expanded case-mix + other anti-diabetes medications analyses adjusted for expanded case-mix covariates, plus insulin and metformin. Abbrev.: CKD, chronic kidney disease; DPP4i, dipeptidyl peptidase-4 inhibitor; GLP1a, glucagon-like peptide-1 receptor agonist; SGLT2i, sodium-glucose cotransporter-2 inhibitor.

In the overall cohort, compared to DPP4i use, SGLT2i and GLP1a use were not associated with higher risk of amputation: aHRs (95% CIs) 0.94 (0.67, 1.31) and 1.19 (0.98, 1.45), respectively (Fig. 3 and Supplementary Table S16). Similarly, in the CKD cohort, SGLT2i and GLP1a use were not associated with higher risk of amputation (ref: DPP4i use). However, in the non-CKD cohort, whereas SGLT2i was not associated with higher risk of amputation (aHRs [95% CIs] 1.13 [0.70, 1.82]), GLP1a was associated with higher risk (ref: DPP4i use): aHR (95% CI) 1.52 (1.12, 2.07). In PS-matched analyses, we observed a similar pattern of findings in the overall, non-CKD, and CKD cohorts (Supplementary Table S13). In analyses examining granular CKD subcategories, SGLT2i and GLP1a were not associated with higher risk of amputation in stages 1–2 and 3 CKD. However, among patients with stages 4–5 CKD GLP1a vs. DPP4i use was associated with lower risk of amputation (Supplementary Table S14).

### SGLT2i vs. GLP1a vs. DPP4i and risk of diabetic ketoacidosis

**Fig. 4:**
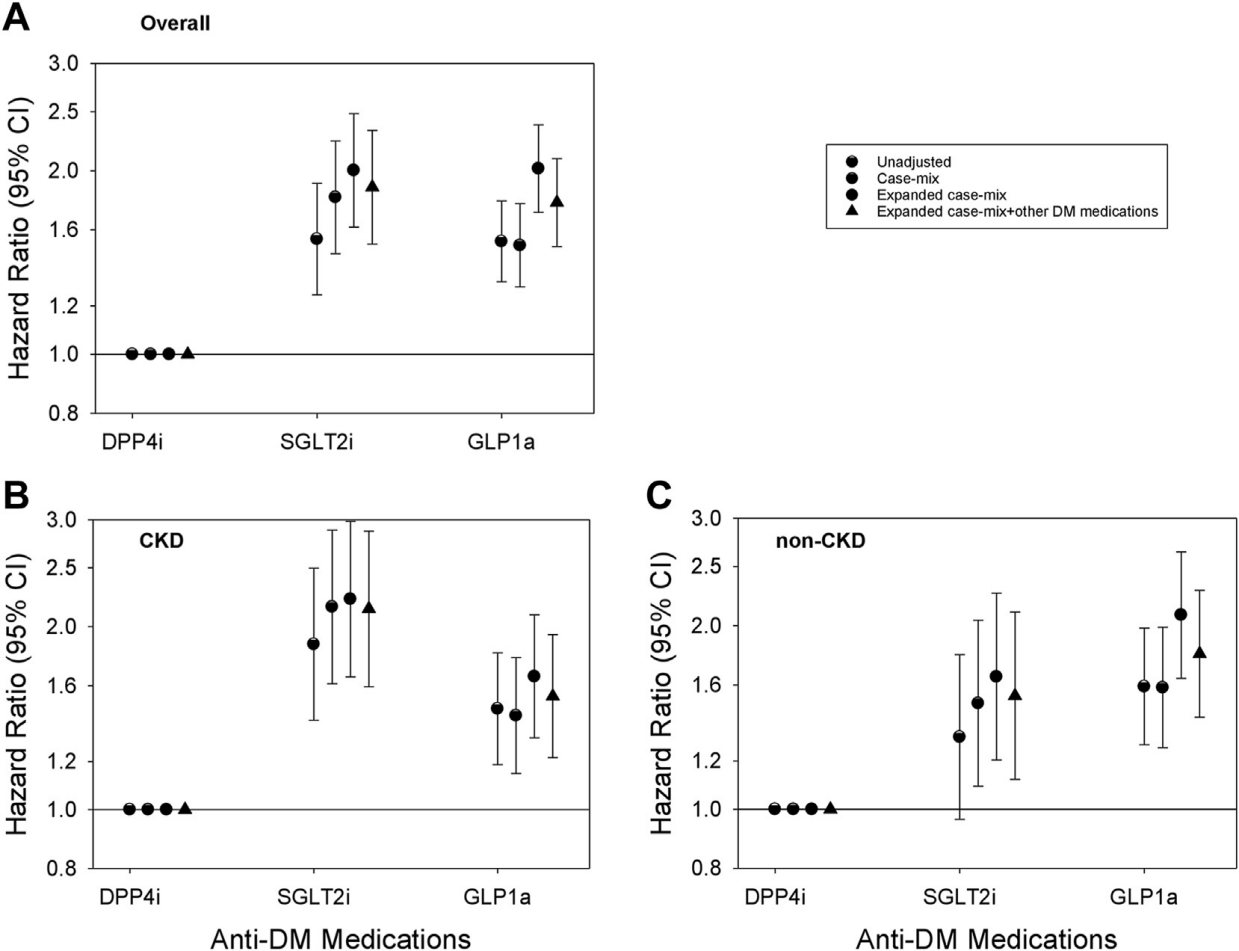
**Association of SGLT2i vs. GLP1a vs. DPP4i medication use with risk of diabetic ketoacidosis in the overall cohort (Panel A; N = 91,684), CKD cohort (Panel B; N = 43,697), and non-CKD cohort (Panel C; N = 47,987).** Each plot shows hazard ratios and their 95% CIs estimated using Cox regression models incrementally adjusted with the following covariates: (1) Unadjusted analyses; (2) Case-mix analyses adjusted for age, sex, race, ethnicity, myocardial infarction, congestive heart failure, cardiovascular disease, and Charlson Comorbidity Index; (3) Expanded case-mix analyses adjusted for case-mix covariates, plus estimated glomerular filtration rate, urine albumin-to-creatinine ratio, serum albumin, body mass index, and glycated hemoglobin; and (4) Expanded case-mix + other anti-diabetes medications analyses adjusted for expanded case-mix covariates, plus insulin and metformin. Abbrev.: CKD, chronic kidney disease; DPP4i, dipeptidyl peptidase-4 inhibitor; GLP1a, glucagon-like peptide-1 receptor agonist; SGLT2i, sodium-glucose cotransporter-2 inhibitor.

In the overall cohort, compared with DPP4i use, both SGLT2i and GLP1a use were each associated with higher risk of DKA: aHRs (95% CIs) 1.88 (1.52, 2.33) and 1.77 (1.50, 2.09), respectively (Fig. 4 and Supplementary Table S17). A similar pattern of findings was observed in the CKD and non-CKD cohorts. In PS-matched analyses, we observed a similar pattern of findings in the non-CKD and CKD cohorts, except SGLT2i vs. DPP4i use showed similar risk of DKA in the non-CKD cohort and GLP1a vs. DPP4i showed similar risk in the non-CKD and CKD cohorts (Supplementary Table S13). In analyses examining granular CKD subcategories, SGLT2i use was associated with higher risk of DKA in patients with stages 1–2 and 3 CKD, and GLP1a use was associated with higher risk of DKA in those with stages 1–2 (Supplementary Table S14).

## Discussion

In this analysis of a large, contemporary national cohort of US Veterans, we found that incident users of SGLT2i had lower risk of infection-related hospitalization as compared with DPP4i users in the overall cohort, as well in the CKD and non-CKD cohorts. When examining cause-specific infection hospitalizations, we also observed that SGLT2i use was associated with lower to similar risk of genitourinary infection hospitalizations in the overall, CKD, and non-CKD cohorts. In sensitivity analyses, while SGLT2i use was not associated with heightened risk of lower extremity amputations nor short-term AKI risk (i.e., within two-days of prescription) vs. DPP4i use, we did observe higher long-term AKI risk (i.e., within 30-, 60- and 90-days of prescription) among SGLT2i and GLP1a users in the overall, CKD, and non-CKD cohorts. This overall pattern of findings was robust across multiple secondary analyses as well as sensitivity analyses using multivariable adjustment and PS-matching to account for confounders.

To our knowledge, this is the first real-world study comparing the risk of adverse events among incident users of SGLT2i vs. GLP1a vs. DPP4i in a large national cohort of patients with vs. without CKD. Recent published clinical trial data in the CKD population have shown the potent effects of SGLT2i on reducing the risk of eGFR decline, ESKD, and renal-/cardiovascular-related mortality^13^^,^^14^ and has been endorsed as first-line diabetes therapy across various practice guidelines.^15^^,^^16^ However, there remains under-utilization of this medication class,^20^ which may in part be due to ongoing debate regarding its safety profile based on mixed data from clinical trials and observational studies.^7^^,^^10^, ^11^, ^12^, ^13^, ^14^^,^^17^ For example, the potential risks of genitourinary infections have been a major concern of SGLT2i based on increased genitourinary infection/urosepsis in the EMPA-REG trial,^12^ serious genitourinary infections in the DECLARE TIMI 58 trial,^11^ and genitourinary infection in the CANVAS trial^10^ among the general population, via the glycosuric effects of this drug. However, in trials specific to the CKD population, while a higher risk of genitourinary infections was reported in the CREDENCE trial,^14^ albeit at a low frequency, this was not observed in the DAPA-CKD trial,^13^ presumably due to attenuation of glycosuria in the context of kidney dysfunction. In our current study, we found that, compared with DPP4i use, SGLT2i use was in fact associated with lower risk of genitourinary infection hospitalizations (including urinary tract infections) and infection-related hospitalizations from any cause in the overall, CKD, and non-CKD cohorts over an intermediate-to-long period, suggesting a relatively safe profile of SGLT2i with respect to moderate-to-severe genitourinary infection risk necessitating medical attention. Yet it also bears mention that the discrepancy observed between the aforementioned trials vs. our real-world data may relate to differences in how genitourinary infections were ascertained (i.e., adjudicated events reported in clinical trials vs. diagnostic codes assessed from hospitalization records). Hence, further studies are needed to confirm our findings and to more precisely define SGLT2i-related genitourinary infection risk in the CKD and non-CKD populations.

Another noteworthy finding of our study was the lack of SGLT2i-related amputation risk as compared with DPP4i use in the overall, CKD, and non-CKD cohorts. While general population data from the CANVAS trial demonstrated higher risk of lower extremity amputations (i.e., transmetatarsal and toe amputations^17^) with SGLT2i use,^7^^,^^10^ in CKD trials (i.e., CREDENCE, DAPA-CKD) rates of lower limb amputation were similar across SGLT2i and placebo groups. In contrast, in PS-matched analyses GLP1a vs. SGLT2i use was associated with similar risk of amputation in overall and non-CKD cohorts but higher risk in those with CKD. While our findings are consistent with pooled analyses of low-to-average atherogenic risk populations,^36^ it is possible that our disparate findings in the CKD cohort may relate to the higher amputation risk observed in Veterans.^37^ We also found that GLP1a vs. DPP4i use was associated with higher risk of amputation in those without CKD. Given that few studies have directly compared GLP1a vs. DPP4i use on amputation risk, further confirmatory studies are needed.

When examining AKI-risk within varying time intervals following SGLT2i vs. GLP1a vs. DPP4i initiation, whereas SGLT2i and GLP1a initiation were not associated with short-term (i.e., within 48-hours of prescription) AKI risk, we observed that use of these medications were associated with AKI across long-term time intervals (i.e., within 30-, 60- or 90-days of prescription) in the overall, CKD, and non-CKD cohorts. While the SGLT2i trials in the general and CKD populations did not demonstrate higher risk of AKI as compared with placebo, greater volume depletion was observed in the SGLT2i arms of both the CREDENCE^14^ and DAPA-CKD trials^13^ in the CKD population and in some trials of the general population (i.e., CANVAS^10^), likely due to glucosuria-induced osmotic diuresis and natriuresis. While the SGLT2i-associated diuretic effect has been postulated to wane over time with subsequent volume stabilization,^17^^,^^18^ our real-world data corroborate recommendations for increased vigilance with respect to ongoing monitoring and correction of volume depletion, as well as judicious use of diuretics and other anti-hypertensive agents^18^^,^^38^ in the context of SGLT2i prescription. Similarly, there have been postmarketing reports and cases of AKI reported following the initiation of GLP1a, presumably due to volume depletion ensuing from the gastrointestinal sequelae of GLP1a (i.e., nausea, vomiting, diarrhea).^39^ Further studies are needed to determine the underlying mechanisms of GLP1a-related AKI events.

Notably, we also identified a heightened risk of DKA associated with SGLT2i and GLP1a use in the overall, CKD, and non-CKD cohorts. In the general population, higher risk of DKA was observed in some (i.e., DECLARE TIMI 58^11^) but not all (i.e., EMPA REG OUTCOME,^12^ CANVAS^10^) trials. Similarly, mixed findings have been reported in the CKD trials, with CREDENCE showing higher SGLT2i-associated DKA risk,^14^ yet DAPA-CKD demonstrating no cases with SGLT2i use.^13^ Given that SGLT2i may theoretically induce euglycemic DKA vis-à-vis increased fatty acid oxidation, glucagon release, and decreased insulin secretion,^8^ particularly in diabetic kidney disease patients who may be at greater underlying risk due to restricted carbohydrate intake and deficient insulin production and/or secretion,^4^, ^5^, ^6^ our findings support recommendations for maintenance and/or cautious modulation of insulin regimens, consideration of temporary cessation of SGLT2i during acute illness,^38^ and careful monitoring of symptoms and signs (i.e., blood/urine ketone monitoring) to mitigate DKA risk in this population.^8^ While the mechanistic link between GLP1a use and heightened DKA risk is not clear, the gastrointestinal-related symptoms of these drugs could be a predisposing factor. Alternatively, there have been reports of DKA following GLP1a initiation, particularly when prescribed in the absence of insulin, and/or when concomitant insulin was rapidly reduced or discontinued.^40^^,^^41^ Further research is needed to determine the real-world incidence of mechanistic underpinnings of GLP1a-related DKA.

The strengths of our study include its examination of a large national cohort of patients with and without CKD with comprehensive capture of detailed clinical data, including longitudinal laboratory and prescription information^1^^,^^21^^,^^42^; reduced confounding by differential health care access and nonuniform medical care within the VA healthcare system^1^^,^^21^^,^^34^^,^^42^; use of a rigorous incident/new user medication design^43^; comparison of SGLT2i with other novel first- and second-line anti-diabetic medications; and availability of long-term follow up data to identify short-, intermediate-, and long-term adverse events. However, several limitations of our study should be acknowledged. First, our study consisted of patients who were largely male, of older age, of higher comorbidity burden, and of non-Hispanic White race, which may limit generalizability of our findings and precluded granular subgroup analyses across different age groups and race/ethnicities. Second, our primarily analyses of infection-related complications (including genitourinary infection) solely focused on serious infection events necessitating medical attention (i.e., hospitalization), and did not take into account milder infections that were managed in the outpatient setting. Third, while our database had detailed availability of prescription data, we cannot confirm that patients were compliant with dispensed medication regimens, nor were we able to examine the safety outcomes of individual medications within the SGLT2i, DPP4i, and GLP1a classes, and therefore did not granularly examine drug doses. While there is emerging data showing a class effect between individual SGLT2i and cardiovascular outcomes,^44^ future studies are needed to determine the impact of individual medications within these anti-glycemic medication categories with safety outcomes. Furthermore, while the present study examined anti-diabetic medications as monotherapies, there is increasing data examining the impact of combination treatment, namely combined SGLT2i and GLP1a use, showing potential benefit on cardiovascular, kidney disease, and all-cause mortality outcomes in patients with type 2 diabetes and albuminuria^45^; future studies are needed to determine the comparative effectiveness of combination therapy in patients with CKD with diabetes. Fourth, due to data limitations, we were not able to take into account duration of diabetes as a potential confounder. Finally, given the observational nature of our study, we cannot exclude the possibility of residual confounding, and our findings cannot confirm a causal relationship between SGLT2i, GLP1a, or DPP4i with the outcomes of interest.

In conclusion, in this real-world analysis of US Veterans, we found that SGLT2i use was associated with lower risk of infection-related hospitalizations, including those related to genitourinary infection, among patients with and without CKD. Given that SGLT2i are considered first-line therapy for diabetes, as well as our findings demonstrating a relatively safe profile of SGLT2i with respect to moderate-to-severe infections over an intermediate-to-long follow-up period, further strategies and research are needed to optimize uptake of these medications in the real-world settings and to identify which subpopulations will most benefit from their utilization.

## Contributors

YN, CPK, and CMR were involved in the conception, design, and conduct of the study and interpretation of the results. YN conducted the data analysis of the study. YN and CMR wrote the first draft of the manuscript, and YN, CPK, ASY, KS, YM, SS, FT, ANA, ES, KKZ, and CMR edited, reviewed, and approved the final version of the manuscript. CPK and CMR had full access to all the data in the study and take responsibility for the integrity of the data and the accuracy of the data analysis.

## Data sharing statement

Due to the nature of the research, due to restrictions (i.e., data containing information that could compromise the privacy of research participants), supporting data is not available.

## Declaration of interests

CPK is supported by grant No. I01HX002680 from the VA Health Services Research and Development Service and has received royalties for two chapters from UpToDate and co-editing textbook on renal nutrition from Springer; consulting fees from Abbot, Akebia, AstraZeneca, Bayer, Boehringer Ingelheim, Cara Therapeutics, CSL Vifor, Eli Lilly, GSK, Pharmacosmos, ProKidney, CSL Behring, Renibus, and Rockwell; payment from Takeda for expert testimony; travel support from AstraZeneca, Bayer, and Boehringer Ingelheim; participation on a Data Safety Monitoring Board or Advisory Board of AstraZeneca (DIALIZE-O trial executive committee, GRAZE trial scientific committee) and Bayer (CardioRenal council member, FOUNTAIN Program Executive Advisory Committee Co-chair); and is President-Elect of the International Society of Renal Nutrition and Metabolism, which are unrelated to this study and manuscript.

SS is funded by the NIH/NIMHD and CDC, which are unrelated to this study and manuscript.

FT is supported by grant No. 1I01HX002680-01A1 from the VA the VA Health Services Research and Development Service through IPA (5 U.S.C. 3371–3376) with University of Tennessee Health Science Center.

AA has served as primary or co-investigator of clinical trials sponsored by NIH/NIAID, NeuroRx Pharma, Pulmotect, Blade Therapeutics, Novartis, Takeda, Humanigen, Eli Lilly, PTC Therapeutics, Octapharma, Fulcrum Therapeutics, and Alexion; and as a speaker and/or consultant for Pfizer, Salix, Alexion, AstraZeneca, Bayer, Ferring, Seres, Spero, Eli Lilly, Nova Nordisk, Gilead, Renibus, GSK, Dexcom, Reprieve, HeartRite, Aseptiscope, which are unrelated to this study and manuscript.

KKZ is supported by National Institute of Health and Veterans Administration; received honoraria and/or support from Ardelyx, American Society of Nephrology, DaVita, Fresenius, GSK, Haymarket Media, International Society of Renal Nutrition & Metabolism, Kabi, National Institutes of Health, National Kidney Foundations, VA, Vifor/CSL, and UpToDate, which are unrelated to this study and manuscript.

None of the other authors declare competing interests.

## Appendix ASupplementary data

Supplementary Figs. S1–S6 and Tables S1–S17

AntiDMMed_Safety_LanReg_CL
