# Supplementary material for: Safety of SGLT2 inhibitors, DPP-4 inhibitors, and GLP-1 receptor agonists in US veterans with and without chronic kidney disease: a population-based study
Source: Lancet Reg Health Am. 2024 Jun 18;36:100814. doi: 10.1016/j.lana.2024.100814 (PMC11237921; doi:10.1016/j.lana.2024.100814)

Title: Safety of SGLT2 Inhibitors, DPP-4 Inhibitors, and GLP-1 Receptor Agonists in US Veterans with and without Chronic Kidney Disease: A Population-Based Study

ONLINE-ONLY SUPPLEMENTARY MATERIAL

TABLE OF CONTENTS

Supplementary Table 1. Individual medications within anti-diabetic medication classes (Panel A), and diagnostic and procedure codes used to ascertain lower extremity amputation and diabetic ketoacidosis events (Panel B). *Abbrev.: DPP4i, dipeptidyl peptidase-4 inhibitor; GLP1a, glucagon-like peptide-1 receptor agonist; SGLT2, sodium-glucose cotransporter-2 inhibitor.*

Supplementary Table 2. Baseline characteristics according to SGLT2i, DPP4i, and GLP1a use in the overall, CKD, and non-CKD cohorts in the acute kidney injury analyses. *Abbrev.: CHF, congestive heart failure; CVD, cardiovascular disease; DPP4i, dipeptidyl peptidase-4 inhibitor; GLP1a, glucagon-like peptide-1 receptor agonist; MI, myocardial infarction; SGLT2i, sodium-glucose cotransporter-2 inhibitor; UACR, urine albumin-to-creatinine ratio; AKI, acute kidney injury.*

Supplementary Table 3. Baseline characteristics according to SGLT2i, DPP4i, and GLP1a use in the overall, CKD, and non-CKD cohorts in the amputation analyses. *Abbrev.: CHF, congestive heart failure; CVD, cardiovascular disease; DPP4i, dipeptidyl peptidase-4 inhibitor; GLP1a, glucagon-like peptide-1 receptor agonist; MI, myocardial infarction; SGLT2i, sodium-glucose cotransporter-2 inhibitor; UACR, urine albumin-to-creatinine ratio; AKI, acute kidney injury.*

Supplementary Table 4. Baseline characteristics according to SGLT2i, DPP4i, and GLP1a use in the overall, CKD, and non-CKD cohorts in the diabetic ketoacidosis analyses. Continuous variables are presented as means (SDs) or medians (IQRs) as dictated by data type and categorical variables are presented as column percentages. *Abbrev.: CHF, congestive heart failure; CVD, cardiovascular disease; DPP4i, dipeptidyl peptidase-4 inhibitor; GLP1a, glucagon-like peptide-1 receptor agonist; MI, myocardial infarction; SGLT2i, sodium-glucose cotransporter-2 inhibitor; UACR, urine albumin-to-creatinine ratio; AKI, acute kidney injury.*

Supplementary Table 5. Descriptives of death and end-stage kidney disease events among the time to first infection-related hospitalization cohort.

Supplementary Table 6. Association of SGLT2i vs. GLP1a vs. DPP4i medication use with time to the first infection-related hospitalization in the overall cohort (Panel A; N=92,269), CKD cohort (Panel B; N=44,036), and non-CKD cohort (Panel C; N=48,233). *Each plot shows hazard ratios and their 95% CIs estimated using Cox regression models incrementally adjusted with the following covariates: (1) Unadjusted analyses; (2) Case-mix analyses adjusted for age, sex, race, ethnicity, myocardial infarction, congestive heart failure, cardiovascular disease, and Charlson Comorbidity Index; (3) Expanded case-mix analyses adjusted for case-mix covariates, plus estimated glomerular filtration rate, urine albumin-to-creatinine ratio, serum albumin, body mass index, and glycated hemoglobin; and (4) Expanded case-mix+other anti-diabetes medications analyses adjusted for expanded case-mix covariates, plus insulin and metformin. Abbrev.: DPP4i, dipeptidyl peptidase-4 inhibitor; GLP1a, glucagon-like peptide-1 agonist; SGLT2i, sodium-glucose cotransporter-2 inhibitor.*

Supplementary Table 7. Comparison of results from propensity score (PS) matched vs. non-PS matched analyses incorporating expanded case-mix+laboratory+other DM medication covariates examining associations of SGLT2i vs. GLP1a vs. DPP4i medication use with time to first infection-related hospitalization, frequency of infection-related hospitalization, time to first genitourinary infection hospitalization, and frequency of genitourinary infection hospitalization. *Each plot shows hazard ratios and their 95% CIs estimated using Cox regression models incrementally adjusted with the following covariates: (4) Expanded case-mix+other anti-diabetes medications analyses adjusted for expanded case-mix covariates, plus insulin and metformin. Abbrev.: DM, diabetes mellitus; DPP4i, dipeptidyl peptidase-4 inhibitor; GLP1a, glucagon-like peptide-1 receptor agonist; PSM, propensity score match; SGLT2i, sodium-glucose cotransporter-2 inhibitor.*

Supplementary Table 8. Subgroup analysis of association of SGLT2i vs. GLP1a vs. DPP4i medication use with risk of time to first acute kidney injury, amputation, and diabetic ketoacidosis in expanded case-mix+laboratory+other DM medication adjusted models. *Each plot shows hazard ratios and their 95% CIs estimated using Cox regression models incrementally adjusted with the following covariates: (4) Expanded case-mix+other anti-diabetes medications analyses adjusted for expanded case-mix covariates, plus insulin and metformin. Abbrev.: AKI, acute kidney injury; DM, diabetes mellitus; DKA, diabetic ketoacidosis; DPP4i, dipeptidyl peptidase-4 inhibitor; GLP1a, glucagon-like peptide-1 receptor agonist; SGLT2i, sodium-glucose cotransporter-2 inhibitor.*

Supplementary Table 9. Association of SGLT2i vs. GLP1a vs. DPP4i medication use with frequency of infection-related hospitalizations in the overall cohort (Panel A; N=92,269), CKD cohort (Panel B; N=44,036), and non-CKD cohort (Panel C; N=48,233). *Each plot shows hazard ratios and their 95% CIs estimated using Cox regression models incrementally adjusted with the following covariates: (1) Unadjusted analyses; (2) Case-mix analyses adjusted for age, sex, race, ethnicity, myocardial infarction, congestive heart failure, cardiovascular disease, and Charlson Comorbidity Index; (3) Expanded case-mix analyses adjusted for case-mix covariates, plus estimated glomerular filtration rate, urine albumin-to-creatinine ratio, serum albumin, body mass index, and glycated hemoglobin; and (4) Expanded case-mix+other anti-diabetes medications analyses adjusted for expanded case-mix covariates, plus insulin and metformin. Abbrev.: DPP4i, dipeptidyl peptidase-4 inhibitor; GLP1a, glucagon-like peptide-1 receptor agonist; SGLT2i, sodium-glucose cotransporter-2 inhibitor.*

Supplementary Table 10. Association of SGLT2i vs. GLP1a vs. DPP4i medication use with time to the first genitourinary infection hospitalization in the overall cohort (Panel A; N=92,269), CKD cohort (Panel B; N=44,036), and non-CKD cohort (Panel C; N=48,233). *Each plot shows hazard ratios and their 95% CIs estimated using Cox regression models incrementally adjusted with the following covariates: (1) Unadjusted analyses; (2) Case-mix analyses adjusted for age, sex, race, ethnicity, myocardial infarction, congestive heart failure, cardiovascular disease, and Charlson Comorbidity Index; (3) Expanded case-mix analyses adjusted for case-mix covariates, plus estimated glomerular filtration rate, urine albumin-to-creatinine ratio, serum albumin, body mass index, and glycated hemoglobin; and (4) Expanded case-mix+other anti-diabetes medications analyses adjusted for expanded case-mix covariates, plus insulin and metformin. Abbrev.: DPP4i, dipeptidyl peptidase-4 inhibitor; GLP1a, glucagon-like peptide-1 receptor agonist; SGLT2i, sodium-glucose cotransporter-2 inhibitor.*

Supplementary Table 11. Association of SGLT2i vs. GLP1a vs. DPP4i medication use with frequency of genitourinary infection hospitalizations in the overall cohort (Panel A; N=92,269), CKD cohort (Panel B; N=44,036), and non-CKD cohort (Panel C; N=48,233). *Each plot shows hazard ratios and their 95% CIs estimated using Cox regression models incrementally adjusted with the following covariates: (1) Unadjusted analyses; (2) Case-mix analyses adjusted for age, sex, race, ethnicity, myocardial infarction, congestive heart failure, cardiovascular disease, and Charlson Comorbidity Index; (3) Expanded case-mix analyses adjusted for case-mix covariates, plus estimated glomerular filtration rate, urine albumin-to-creatinine ratio, serum albumin, body mass index, and glycated hemoglobin; and (4) Expanded case-mix+other anti-diabetes medications analyses adjusted for expanded case-mix covariates, plus insulin and metformin. Abbrev.: DPP4i, dipeptidyl peptidase-4 inhibitor; GLP1a, glucagon-like peptide-1 receptor agonist; SGLT2i, sodium-glucose cotransporter-2 inhibitor.*

Supplementary Table 12. Association of SGLT2i vs. GLP1a vs. DPP4i medication use with risk of acute kidney injury in the overall cohort, CKD cohort, and non-CKD cohort with serum creatinine data.

Supplementary Table 13. Comparison of results from propensity score (PS) matched vs. non-PS matched analyses incorporating expanded case-mix+laboratory+other DM medication covariates examining associations of SGLT2i vs. GLP1a vs. DPP4i medication use with risk of time to first acute kidney injury, amputation, and diabetic ketoacidosis. *Each plot shows hazard ratios and their 95% CIs estimated using Cox regression models incrementally adjusted with the following covariates: (4) Expanded case-mix+other anti-diabetes medications analyses adjusted for expanded case-mix covariates, plus insulin and metformin. Abbrev.: AKI, acute kidney injury; DKA, diabetic ketoacidosis; DM, diabetes mellitus; DPP4i, dipeptidyl peptidase-4 inhibitor; GLP1a, glucagon-like peptide-1 receptor agonist; PSM, propensity score match; SGLT2i, sodium-glucose cotransporter-2 inhibitor.*

Supplementary Table 14. Subgroup analysis of association of SGLT2i vs. GLP1a vs. DPP4i medication use with risk of time to first infection-related hospitalization, frequency of infection-related hospitalization, time to first genitourinary infection hospitalization, and frequency of genitourinary infection hospitalization in expanded case-mix+laboratory+other DM medication adjusted models. *Each plot shows hazard ratios and their 95% CIs estimated using Cox regression models incrementally adjusted with the following covariates: (4) Expanded case-mix+other anti-diabetes medications analyses adjusted for expanded case-mix covariates, plus insulin and metformin. Abbrev.: DPP4i, dipeptidyl peptidase-4 inhibitor; GLP1a, glucagon-like peptide-1 receptor agonist; SGLT2i, sodium-glucose cotransporter-2 inhibitor.*

Supplementary Table 15. Drug discontinuation according to presence vs. absence of acute kidney injury (AKI) within 48-hours of initial drug prescription among patients with serum creatinine data values (N=5,715). *Abbrev.: AKI, acute kidney injury; DPP4i, dipeptidyl peptidase-4 inhibitor; GLP1a, glucagon-like peptide-1 receptor agonist; SGLT2i, sodium-glucose cotransporter-2 inhibitor.*

Supplementary Table 16. Association of SGLT2i vs. GLP1a vs. DPP4i medication use with risk of lower extremity amputation in the overall cohort (Panel A; N=88,281), CKD cohort (Panel B; N=41,917), non-CKD cohort (Panel C; N=46,364). *Each plot shows hazard ratios and their 95% CIs estimated using Cox regression models incrementally adjusted with the following covariates: (1) Unadjusted analyses; (2) Case-mix analyses adjusted for age, sex, race, ethnicity, myocardial infarction, congestive heart failure, cardiovascular disease, and Charlson Comorbidity Index; (3) Expanded case-mix analyses adjusted for case-mix covariates, plus estimated glomerular filtration rate, urine albumin-to-creatinine ratio, serum albumin, body mass index, and glycated hemoglobin; and (4) Expanded case-mix+other anti-diabetes medications analyses adjusted for expanded case-mix covariates, plus insulin and metformin. Abbrev.: DPP4i, dipeptidyl peptidase-4 inhibitor; GLP1a, glucagon-like peptide-1 receptor agonist; SGLT2i, sodium-glucose cotransporter-2 inhibitor.*

Supplementary Table 17. Association of SGLT2i vs. GLP1a vs. DPP4i medication use with risk of diabetic ketoacidosis in the overall cohort (Panel A; N=91,684), CKD cohort (Panel B; N=43,697), and non-CKD cohort (Panel C; N=47,987). *Each plot shows hazard ratios and their 95% CIs estimated using Cox regression models incrementally adjusted with the following covariates: (1) Unadjusted analyses; (2) Case-mix analyses adjusted for age, sex, race, ethnicity, myocardial infarction, congestive heart failure, cardiovascular disease, and Charlson Comorbidity Index; (3) Expanded case-mix analyses adjusted for case-mix covariates, plus estimated glomerular filtration rate, urine albumin-to-creatinine ratio, serum albumin, body mass index, and glycated hemoglobin; and (4) Expanded case-mix+other anti-diabetes medications analyses adjusted for expanded case-mix covariates, plus insulin and metformin. Abbrev.: DPP4i, dipeptidyl peptidase-4 inhibitor; GLP1a, glucagon-like peptide-1 receptor agonist; SGLT2i, sodium-glucose cotransporter-2 inhibitor.*

Supplementary Figure 1. Study cohort creation for infection-related hospitalization analyses. *Abbrev.: DPP4i, dipeptidyl peptidase-4 inhibitor; GLP1a, glucagon-like peptide-1 receptor agonist; SGLT2i, sodium-glucose cotransporter-2 inhibitor.*

Supplementary Figure 2. Study cohort creation for acute kidney injury analyses *Abbrev.: DPP4i, dipeptidyl peptidase-4 inhibitor; GLP1a, glucagon-like peptide-1 receptor agonist; SGLT2i, sodium-glucose cotransporter-2* inhibitor.

Supplementary Figure 3. Study cohort creation for amputation analyses. *Abbrev.: DPP4i, dipeptidyl peptidase-4 inhibitor; GLP1a, glucagon-like peptide-1 receptor agonist; SGLT2i, sodium-glucose cotransporter-2 inhibitor.*

Supplementary Figure 4. Study cohort creation for diabetic ketoacidosis analyses. *Abbrev.: DPP4i, dipeptidyl peptidase-4 inhibitor; GLP1a, glucagon-like peptide-1 receptor agonist; SGLT2i, sodium-glucose cotransporter-2 inhibitor.*

Supplementary Figure 5. Association of SGLT2i vs. GLP1a vs. DPP4i medication use with time to the first genitourinary infection hospitalization in the overall cohort (Panel A; N=92,269), CKD cohort (Panel B; N=44,036), and non-CKD cohort (Panel C; N=48,233). *Each plot shows hazard ratios and their 95% CIs estimated using Cox regression models incrementally adjusted with the following covariates: (1) Unadjusted analyses; (2) Case-mix analyses adjusted for age, sex, race, ethnicity, myocardial infarction, congestive heart*

failure, cardiovascular disease, and Charlson Comorbidity Index; (3) Expanded case-mix analyses adjusted for case-mix covariates, plus estimated glomerular filtration rate, urine albumin-to-creatinine ratio, serum albumin, body mass index, and glycated hemoglobin; and (4) Expanded case-mix+other anti-diabetes medications analyses adjusted for expanded case-mix covariates, plus insulin and metformin. Abbrev.: DPP4i, dipeptidyl peptidase-4 inhibitor; GLP1a, glucagon-like peptide-1 receptor agonist; SGLT2i, sodium-glucose cotransporter-2 inhibitor.

Supplementary Figure 6. Association of SGLT2i vs. GLP1a vs. DPP4i medication use with frequency of genitourinary infection hospitalizations in the overall cohort (Panel A; N=92,269), CKD cohort (Panel B; N=44,036), and non-CKD cohort (Panel C; N=48,233). *Each plot shows hazard ratios and their 95% CIs estimated using Cox regression models incrementally adjusted with the following covariates: (1) Unadjusted analyses; (2) Case-mix analyses adjusted for age, sex, race, ethnicity, myocardial infarction, congestive heart failure, cardiovascular disease, and Charlson Comorbidity Index; (3) Expanded case-mix analyses adjusted for case-mix covariates, plus estimated glomerular filtration rate, urine albumin-to-creatinine ratio, serum albumin, body mass index, and glycated hemoglobin; and (4) Expanded case-mix+other anti-diabetes medications analyses adjusted for expanded case-mix covariates, plus insulin and metformin. Abbrev.: DPP4i, dipeptidyl peptidase-4 inhibitor; GLP1a, glucagon-like peptide-1 receptor agonist; SGLT2i, sodium-glucose cotransporter-2 inhibitor.*

Supplementary Table 1. Individual medications within anti-diabetic medication classes (Panel A), and diagnostic and procedure codes used to ascertain lower extremity amputation and diabetic ketoacidosis events (Panel B).

MEDICATION CLASS	INDIVIDUAL MEDICATIONS
**SGLT2i class**	Canagliflozin<br>Dapagliflozin<br>Empagliflozin
**DPP4i class**	Alogliptin<br>Linagliptin<br>Saxagliptin<br>Sitagliptin<br>Vildagliptin
**GLP1a class**	Albiglutide<br>Dulaglutide<br>Exenatide<br>Liraglutide<br>Lixisenatide<br>Semaglutide

CLINICAL CONDITION	ICD9 CODE	ICD10 CODE	CPT CODE
**LOWER EXTREMITY AMPUTATION**	84.1x, 84.91		27295, 27590-92, 27598, 27880-82, 27888-89, 28800, 28805
**DIABETIC KETOACIDOSIS**	2491, 24910, 24911, 2501, 25010, 25011, 25012, 25013	E081, E0810, E0811, E091, E0910, E0911, E101, E1010, E1011, E1110, E1111, E131, E1310, E1311	

Abbrev.: DPP4, dipeptidyl peptidase-4 inhibitor; GLP1, glucagon-like peptide-1 receptor agonist; SGLT2, sodium-glucose cotransporter-2 inhibitor.

Supplementary Table 2. Baseline characteristics according to SGLT2i, DPP4i, and GLP1a use in the overall, CKD, and non-CKD cohorts in the acute kidney injury analyses.

	Overall				CKD				Non-CKD			
	Overall	Anti-DM Medication			Overall	Anti-DM Medication			Overall	Anti-DM Medication		
		SGLT2i	DPP4i	GLP1a		SGLT2i	DPP4i	GLP1a		SGLT2i	DPP4i	GLP1a
N (% out of overall/CKD/non-CKD) of participants	92,140 (100)	13,050 (14.2)	62,194 (67.5)	16,896 (18.3)	43,973 (100)	5,551 (12.6)	29,352 (66.8)	9,070 (20.6)	48,167 (100)	7,499 (15.6)	32,842 (68.2)	7,826 (16.2)
Age (years), mean (SD)	68(9)	67(8)	69(10)	65(9)	70(9)	69(8)	72(9)	67(8)	66(9)	66(8)	67(9)	63(9)
Male, N (%)	88,080(96)	12,597(97)	59,683(96)	15,800(94)	89,127(97)	12,702(97)	60,378(97)	16,089(95)	45,545(95)	7,194(96)	31,188(95)	7,163(92)
White race, N (%)	69,949(76)	10,088(77)	47,096(76)	12,765(76)	70,432(76)	10,088(77)	47,423(76)	12,930(77)	36,336(75)	5,797(77)	24,715(75)	5,824(74)
Black race, N (%)	16,652(18)	2,269(17)	11,257(18)	3,126(19)	15,506(17)	2,226(17)	10,395(17)	2,882(17)	9,252(19)	1,322(18)	6,351(19)	1,579(20)
Other race, N (%)	1,810(2)	262(2)	1,242(2)	306(2)	1,907(2)	299(2)	1,284(2)	330(2)	900(2)	135(2)	636(2)	129(2)
Missing race, N (%)	0(0)	0(0)	0(0)	0(0)	0(0)	0(0)	0(0)	0(0)	0(0)	0(0)	0(0)	0(0)
Asian/Pacific Islander race, N (%)	2,125(2)	260(2)	1,463(2)	402(2)	2,309(3)	280(2)	1,621(3)	406(2)	1,023(2)	141(2)	698(2)	184(2)
Hispanic ethnicity, N (%)	7,409(8)	1,188(9)	4,986(8)	1,235(7)	6,422(7)	1,077(8)	4,297(7)	1,079(6)	4,344(9)	730(10)	2,958(9)	656(8)
eGFR, mL/min/1.73 m², mean (SD)	72(21)	76(18)	71(21)	71(23)	59(21)	67(19)	58(20)	58(22)	83(14)	83(14)	83(14)	85(15)
UACR (mg/g), median (IQR)	49 (6,48718)	52 (6,48718)	40 (5,29278)	67 (11,20172)	151 (56,48718)	183 (67,48718)	120 (47,29278)	236 (67,20172)	8 (0,30)	8 (0,30)	8 (0,30)	9 (0,30)
Albumin (g/dl), mean (SD)	4.0(0.4)	4.0(0.4)	4.0(0.4)	3.9(0.4)	3.9(0.4)	3.9(0.4)	3.9(0.4)	3.8(0.4)	4.0(0.4)	4.0(0.4)	4.0(0.4)	4.0(0.4)
BMI (kg/m^2), mean (SD)	32.8(6.6)	33.4(6.3)	31.6(6.1)	36.6(7.1)	32.7(6.7)	33.4(6.3)	31.4(6.1)	36.6(7.0)	32.8(6.6)	33.3(6.3)	31.7(6.1)	36.7(7.2)
HbA1c (%), N (%)	8.5(1.6)	8.6(1.4)	8.3(1.6)	9.0(1.6)	8.5(1.6)	8.7(1.4)	8.3(1.5)	9.0(1.5)	8.4(1.6)	8.6(1.4)	8.3(1.7)	8.9(1.6)
MI, N (%)	19,967(22)	3,375(26)	13,087(21)	3,505(21)	24,055(26)	3,776(29)	15,915(26)	4,402(26)	8,487(18)	1,769(24)	5,576(17)	1,142(15)
CHF, N (%)	27,214(30)	3,653(28)	18,521(30)	5,040(30)	34,758(38)	4,380(34)	23,857(38)	6,457(38)	10,626(22)	1,790(24)	7,262(22)	1,574(20)

CVD, N (%)	21,349(23)	2,774(21)	15,402(25)	3,173(19)	25,549(28)	3,207(25)	18,517(30)	3,893(23)	9,156(19)	1,410(19)	6,663(20)	1,083(14)
Charlson Comorbidity Index, mean (SD)	5(3)	5(3)	5(3)	5(3)	6(3)	5(3)	6(3)	6(3)	4(3)	4(3)	5(3)	4(3)
Insulin, N (%)	66,834(73)	10,028(77)	41,227(66)	15,579(92)	70,723(77)	10,586(81)	43,806(70)	15,974(95)	33,082(69)	5,525(74)	20,553(63)	7,004(89)
Metformin, N (%)	88,567(96)	12,721(97)	59,436(96)	16,410(97)	87,218(95)	12,681(97)	58,287(94)	16,248(96)	46,943(97)	7,327(98)	31,928(97)	7,688(98)

CKD was defined as a baseline eGFR <60ml/min/1·73m² (including an eGFR <30ml/min/1·73m²) or baseline albuminuria ≥30mg/g on or before study entry. Continuous variables are presented as means (SDs) or medians (IQRs) as dictated by data type and categorical variables are presented as column percentages. Abbrev.: CHF, congestive heart failure; CVD, cardiovascular disease; DPP4i, dipeptidyl peptidase-4 inhibitor; GLP1a, glucagon-like peptide-1 receptor agonist; MI, myocardial infarction; SGLT2i, sodium-glucose cotransporter-2 inhibitor; UACR, urine albumin-to-creatinine ratio; AKI, acute kidney injury

Supplementary Table 3. Baseline characteristics according to SGLT2i, DPP4i, and GLP1a use in the overall, CKD, and non-CKD cohorts in the amputation analyses.

	Overall				CKD				Non-CKD			
	Overall	Anti-DM Medication			Overall	Anti-DM Medication			Overall	Anti-DM Medication		
		SGLT2i	DPP4i	GLP1a		SGLT2i	DPP4i	GLP1a		SGLT2i	DPP4i	GLP1a
N (% out of overall/CKD/non-CKD) of participants	88,281 (100)	11,601 (13.1)	60,619 (68.7)	16,061 (18.2)	41,917 (100)	4,837 (11.5)	28,537 (68.1)	8,543 (20.4)	46,364 (100)	6,764 (14.6)	32,082 (69.2)	7,518 (16.2)
Age (years), mean (SD)	68(9)	67(8)	69(10)	65(9)	70(9)	69(8)	72(9)	67(8)	66(9)	66(8)	67(9)	63(9)
Male, N (%)	84,416(96)	11,207(97)	58,200(96)	15,009(93)	40,555(97)	4,712(97)	27,708(97)	8,135(95)	43,861(95)	6,495(96)	30,492(95)	6,874(91)
White race, N (%)	67,074(76)	8,977(77)	45,961(76)	12,136(76)	32,045(76)	3,730(77)	21,776(76)	6,539(77)	35,029(76)	5,247(78)	24,185(75)	5,597(74)
Black race, N (%)	15,873(18)	2,002(17)	10,900(18)	2,971(18)	7,021(17)	827(17)	4,739(17)	1,455(17)	8,852(19)	1,175(17)	6,161(19)	1,516(20)
Other race, N (%)	1,743(2)	238(2)	1,207(2)	298(2)	870(2)	115(2)	584(2)	171(2)	873(2)	123(2)	623(2)	127(2)
Missing race, N (%)	0(0)	0(0)	0(0)	0(0)	0(0)	0(0)	0(0)	0(0)	0(0)	0(0)	0(0)	0(0)
Asian/Pacific Islander race, N (%)	2,034(2)	233(2)	1,427(2)	374(2)	1,051(3)	105(2)	747(3)	199(2)	983(2)	128(2)	680(2)	175(2)
Hispanic ethnicity, N (%)	7,064(8)	1,043(9)	4,846(8)	1,175(7)	2,897(7)	394(8)	1,955(7)	548(6)	4,167(9)	649(10)	2,891(9)	627(8)
eGFR (mL/min/1.73 m²), mean (SD)	72(21)	77(18)	71(21)	71(23)	59(21)	68(19)	58(20)	59(22)	83(14)	83(14)	83(14)	85(15)
UACR (mg/g), median ((IQR)	48 (6,48718)	50 (6,48718)	39 (5,29278)	67 (11,20172)	149 (56,48718)	180 (67,48718)	119 (47,29278)	2236 (67,20172)	8 (0,30)	8 (0,30)	8 (0,30)	9 (0,30)
Albumin (g/dl), mean (SD)	4.0(0.4)	4.0(0.4)	4.0(0.4)	3.9(0.4)	3.9(0.4)	3.9(0.4)	3.9(0.4)	3.8(0.4)	4.0(0.4)	4.0(0.4)	4.0(0.4)	4.0(0.4)
BMI (kg/m^2), mean (SD)	32.8(6.6)	33.5(6.3)	31.6(6.1)	36.7(7.1)	32.7(6.7)	33.6(6.3)	31.4(6.1)	36.7(7.0)	32.8(6.6)	33.4(6.3)	31.7(6.1)	36.7(7.2)
HbA1c (%), mean (SD)	8.4(1.6)	8.6(1.4)	8.3(1.6)	9.0(1.6)	8.5(1.6)	8.7(1.4)	8.3(1.5)	9.0(1.5)	8.4(1.6)	8.6(1.4)	8.3(1.7)	8.9(1.6)
MI, N (%)	19,265(22)	3,054(26)	12,890(21)	3,321(21)	11,027(26)	1,420(29)	7,384(26)	2,223(26)	8,238(18)	1,634(24)	5,506(17)	1,098(15)
CHF, N (%)	26,353(30)	3,280(28)	18,266(30)	4,807(30)	16,011(38)	1,642(34)	11,085(39)	3,284(38)	10,342(22)	1,638(24)	7,181(22)	1,523(20)

CVD, N (%)	20,644(23)	2,510(22)	15,122(25)	3,012(19)	11,744(28)	1,225(25)	8,554(30)	1,965(23)	8,900(19)	1,285(19)	6,568(20)	1,047(14)
Charlson Comorbidity Index, mean (SD)	5(3)	5(3)	5(3)	5(3)	6(3)	5(3)	6(3)	6(3)	4(3)	4(3)	5(3)	4(3)
Insulin, N (%)	64,037(73)	8,926(77)	40,289(66)	14,822(92)	32,144(77)	3,925(81)	20,140(71)	8,079(95)	31,893(69)	5,001(74)	20,149(63)	6,743(90)
Metformin, N (%)	84,839(96)	11,316(98)	57,916(96)	15,607(97)	39,660(95)	4,710(97)	26,729(94)	8,221(96)	45,179(97)	6,606(98)	31,187(97)	7,386(98)

CKD was defined as a baseline eGFR <60ml/min/1·73m^2 (including an eGFR <30ml/min/1·73m^2) or baseline albuminuria ≥30mg/g on or before study entry. Continuous variables are presented as means (SDs) or medians (IQRs) as dictated by data type and categorical variables are presented as column percentages. Abbrev.: CHF, congestive heart failure; CVD, Cardiovascular disease; DPP4i, dipeptidyl peptidase-4 inhibitor; GLP1a, glucagon-like peptide-1 receptor agonist; MI, myocardial infarction; SGLT2i, sodium-glucose cotransporter-2 inhibitor; UACR, urine albumin-to-creatinine ratio

Supplementary Table 4. Baseline characteristics according to SGLT2i, DPP4i, and GLP1a use in the overall, CKD, and non-CKD cohorts in the diabetic ketoacidosis analyses.

	Overall				CKD				Non-CKD			
	Overall	Anti-DM Medication			Overall	Anti-DM Medication			Overall	Anti-DM Medication		
		SGLT2i	DPP4i	GLP1a		SGLT2i	DPP4i	GLP1a		SGLT2i	DPP4i	GLP1a
N (% out of overall/CKD/non-CKD) of participants	91,684 (100)	12,885 (14.1)	62,026 (67.7)	16,773 (18.3)	43,697 (100)	5,463 (12.5)	29,254 (66.9)	8,980 (20.6)	47,987 (100)	7,422 (15.5)	32,772 (68.3)	7,793 (16.2)
Age (years), mean (SD)	68(9)	67(8)	69(10)	65(9)	70(9)	69(8)	72(9)	67(8)	66(9)	66(8)	67(9)	63(9)
Male, N (%)	87,645(96)	12,438(97)	59,525(96)	15,682(93)	42,270(97)	5,317(97)	28,403(97)	8,550(95)	45,375(95)	7,121(96)	31,122(95)	7,132(92)
White race, N (%)	69,615(76)	9,967(77)	46,978(76)	12,670(76)	33,398(76)	4,220(77)	22,309(76)	6,869(76)	36,217(75)	5,747(77)	24,669(75)	5,801(74)
Black race, N (%)	16,557(18)	2,231(17)	11,218(18)	3,108(19)	7,356(17)	933(17)	4,887(17)	1,536(17)	9,201(19)	1,298(17)	6,331(19)	1,572(20)
Other race, N (%)	1,798(2)	258(2)	1,237(2)	303(2)	902(2)	125(2)	602(2)	175(2)	896(2)	133(2)	635(2)	128(2)
Missing race, N (%)	0(0)	0(0)	0(0)	0(0)	0(0)	0(0)	0(0)	0(0)	0(0)	0(0)	0(0)	0(0)
Asian/Pacific Islander race, N (%)	2,114(2)	258(2)	1,458(2)	398(2)	1,095(3)	118(2)	762(3)	215(2)	1,019(2)	140(2)	696(2)	183(2)
Hispanic ethnicity, N (%)	7,368(8)	1,171(9)	4,971(8)	1,226(7)	3,043(7)	451(8)	2,019(7)	573(6)	4,325(9)	720(10)	2,952(9)	653(8)
eGFR (mL/min/1.73 m²), mean (SD)	72(21)	76(18)	71(21)	71(23)	59(21)	67(19)	58(20)	59(22)	83(14)	83(14)	83(14)	85(15)
UACR (mg/g), median (IQR)	49 (6,48718)	52 (6,48718)	40 (5,29278)	67 (11,20172)	151 (56,48718)	183 (67,48718)	120 (47,29278)	235 (67,20172)	8 (0,30)	8 (0,30)	8 (0,30)	9 (0,30)
Albumin (g/dl), mean (SD)	4.0(0.4)	4.0(0.4)	4.0(0.4)	3.9(0.4)	3.9(0.4)	3.9(0.4)	3.9(0.4)	3.8(0.4)	4.0(0.4)	4.0(0.4)	4.0(0.4)	4.0(0.4)
BMI (kg/m^2), mean (SD)	32.8(6.6)	33.4(6.3)	31.6(6.1)	36.7(7.1)	32.7(6.7)	33.5(6.3)	31.4(6.1)	36.6(7.0)	32.8(6.6)	33.3(6.3)	31.7(6.1)	36.7(7.2)
HbA1c (%), mean (SD)	8.5(1.6)	8.6(1.4)	8.3(1.6)	9.0(1.6)	8.5(1.6)	8.7(1.4)	8.3(1.5)	9.0(1.5)	8.4(1.6)	8.6(1.4)	8.3(1.7)	8.9(1.6)
MI, N (%)	19,887(22)	3,348(26)	13,065(21)	3,474(21)	11,415(26)	1,585(29)	7,494(26)	2,336(26)	8,472(18)	1,763(24)	5,571(17)	1,138(15)
CHF, N (%)	27,111(30)	3,614(28)	18,492(30)	5,005(30)	16,509(38)	1,835(34)	11,237(38)	3,437(38)	10,602(22)	1,779(24)	7,255(22)	1,568(20)

CVD, N (%)	21,266(23)	2,746(21)	15,370(25)	3,150(19)	12,125(28)	1,346(25)	8,710(30)	2,069(23)	9,141(19)	1,400(19)	6,660(20)	1,081(14)
Charlson Comorbidity Index, mean (SD)	5(3)	5(3)	5(3)	5(3)	6(3)	5(3)	6(3)	6(3)	4(3)	4(3)	5(3)	4(3)
Insulin, N (%)	66,508(73)	9,907(77)	41,135(66)	15,466(92)	33,537(77)	4,431(81)	20,617(70)	8,489(95)	32,971(69)	5,476(74)	20,518(63)	6,977(90)
Metformin, N (%)	88,123(96)	12,560(97)	59,272(96)	16,291(97)	41,358(95)	5,310(97)	27,413(94)	8,635(96)	46,765(97)	7,250(98)	31,859(97)	7,656(98)

CKD was defined as a baseline eGFR <60ml/min/1·73m^2 (including an eGFR <30ml/min/1·73m^2) or baseline albuminuria ≥30mg/g on or before study entry. Continuous variables are presented as means (SDs) or medians (IQRs) as dictated by data type and categorical variables are presented as column percentages. Abbrev.: CHF, congestive heart failure; CVD, Cardiovascular disease; DPP4i, dipeptidyl peptidase-4 inhibitor; GLP1a, glucagon-like peptide-1 receptor agonist; MI, myocardial infarction; SGLT2i, sodium-glucose cotransporter-2 inhibitor; UACR, urine albumin-to-creatinine ratio; DKA, diabetic ketoacidosis.

Supplementary Table 5. Descriptives of death and end-stage kidney disease events among the time to first infection-related hospitalization cohort.

	Overall	Anti-DM Medications		
		SGLT2i	DPP4i	GLP1a
Death, N (%)				
Overall	15,009 (16)	551 (4)	13,055 (21)	1,403 (8)
CKD	8,637 (13)	398 (4)	7,442 (18)	797 (7)
Non-CKD	6,372 (23)	153 (6)	5,613 (28)	606 (10)
End-stage kidney disease, N (%)				
Overall	839 (<1)	14 (<1)	708 (1)	117 (1)
CKD	170 (<1)	3 (<1)	140 (<1)	27 (0)
Non-CKD	669 (2)	11 (<1)	568 (3)	90 (2)

Abbrev.: CKD, chronic kidney disease; DM, diabetes mellitus; DPP4i, dipeptidyl peptidase-4 inhibitor; GLP1a, glucagon-like peptide-1 receptor agonist; SGLT2i, sodium-glucose cotransporter-2 inhibitor.

Supplementary Table 6. Association of SGLT2i vs. GLP1a vs. DPP4i medication use with time to the first infection-related hospitalization in the overall cohort (Panel A; N=92,269), CKD cohort (Panel B; N=44,036), and non-CKD cohort (Panel C; N=48,233).

	Overall			CKD			Non-CKD		
	Anti-DM Medication			Anti-DM Medication			Anti-DM Medication		
**Adjustment model**	**DPP4i**	**SGLT2i**	**GLP1a**	**DPP4i**	**SGLT2i**	**GLP1a**	**DPP4i**	**SGLT2i**	**GLP1a**
N (% out of overall/CKD/non-CKD) of patients	62,243 (67.5)	13,126 (14.2)	16,900 (18.3)	29,382 (66.7)	5,583 (12.7)	9,071 (20.6)	32,861 (68.1)	7,543 (15.6)	7,829 (16.2)
Unadjusted	Reference	0.61(0.55,0.67)	0.87(0.82,0.92)	Reference	0.57(0.50,0.66)	0.83(0.77,0.90)	Reference	0.69(0.61,0.80)	0.89(0.82,0.98)
Case-mix	Reference	0.79(0.71,0.87)	1.10(1.04,1.17)	Reference	0.75(0.65,0.86)	1.03(0.95,1.11)	Reference	0.83(0.72,0.95)	1.18(1.08,1.29)
Expanded case-mix+laboratory	Reference	0.76(0.69,0.84)	1.03(0.97,1.10)	Reference	0.72(0.62,0.82)	0.95(0.87,1.03)	Reference	0.80(0.70,0.92)	1.11(1.01,1.22)
Expanded case-mix+laboratory+other DM meds	Reference	0.74(0.67,0.81)	0.97(0.91,1.03)	Reference	0.70(0.61,0.81)	0.91(0.84,0.99)	Reference	0.77(0.67,0.88)	1.02(0.93,1.13)

Each plot shows hazard ratios and their 95% Cis estimated using Cox regression models incrementally adjusted with the following covariates: (1) Unadjusted analyses; (2) Case-mix analyses adjusted for age, sex, race, ethnicity, myocardial infarction, congestive heart failure, cardiovascular disease, and Charlson Comorbidity Index; (3) Expanded case-mix analyses adjusted for case-mix covariates, plus estimated glomerular filtration rate, urine albumin-to-creatinine ratio, serum albumin, body mass index, and glycated hemoglobin; and (4) Expanded case-mix+other anti-diabetes medications analyses adjusted for expanded case-mix covariates, plus insulin and metformin.
Abbrev.: CKD, chronic kidney disease; DM, diabetes mellitus; DPP4i, dipeptidyl peptidase-4 inhibitor; GLP1a, glucagon-like peptide-1 receptor agonist; SGLT2i, sodium-glucose cotransporter-2 inhibitor.

Supplementary Table 7. Comparison of results from propensity score (PS) matched vs. non-PS matched analyses incorporating expanded case-mix+laboratory+other DM medication covariates examining associations of SGLT2i vs. GLP1a vs. DPP4i medication use with time to first infection-related hospitalization, frequency of infection-related hospitalization, time to first genitourinary infection hospitalization, and frequency of genitourinary infection hospitalization.

		Time to first infection-related hospitalization			Frequency of infection-related hospitalization			Time to first genitourinary infection hospitalization			Frequency of genitourinary infection hospitalization		
		Anti-DM Medication			Anti-DM Medication			Anti-DM Medication			Anti-DM Medication		
		SGLT2i vs. GLP1a	SGLT2i vs. DPP4i	GLP1a vs. DPP4i	SGLT2i vs. GLP1a	SGLT2i vs. DPP4i	GLP1a vs. DPP4i	SGLT2i vs. GLP1a	SGLT2i vs. DPP4i	GLP1a vs. DPP4i	SGLT2i vs. GLP1a	SGLT2i vs. DPP4i	GLP1a vs. DPP4i
**Overall**													
N (% out of overall/CKD/non-CKD) of patients	Non-PS	NA	13,126 (14.2) vs. 62,243 (67.5)	16,900 (18.3) vs. 62,243 (67.5)	NA	13,126 (14.2) vs. 62,243 (67.5)	16,900 (18.3) vs. 62,243 (67.5)	NA	12,995 (14.1) vs. 62,152 (67.5)	16,867 (18.3) vs. 62,152 (67.5)	NA	12,995 (14.1) vs. 62,152 (67.5)	16,867 (18.3) vs. 62,152 (67.5)
	PS	9,108 (9.9) vs. 9,108 (9.9)	11,865 (12.9) vs. 11,865 (12.9)	14,286 (15.5) vs. 14,286 (15.5)	9,108 (9.9) vs. 9,108 (9.9)	11,865 (12.9) vs. 11,865 (12.9)	14,286 (15.5) vs. 14,286 (15.5)	9,041 (9.8) vs. 9,041 (9.8)	11,745 (12.8) vs. 11,745 (12.8)	14,254 (15.5) vs. 14,254 (15.5)	9,041 (9.8) vs. 9,041 (9.8)	11,745 (12.8) vs. 11,745 (12.8)	14,254 (15.5) vs. 14,254 (15.5)
Results	Non-PS	NA	0.74 (0.67,0.81)	0.97 (0.91,1.03)	NA	0.69 (0.64,0.75)	0.97 (0.92,1.02)	NA	0.61 (0.46,0.83)	0.91 (0.77,1.09)	NA	0.55 (0.41,0.72)	0.91 (0.78,1.06)
	PS	0.90 (0.78,1.03)	0.72 (0.64,0.81)	0.92 (0.86,0.99)	0.85 (0.76,0.95)	0.68 (0.62,0.75)	0.91 (0.86,0.97)	0.87 (0.57,1.31)	0.69 (0.48,0.98)	0.80 (0.65,0.98)	0.81 (0.56,1.16)	0.62 (0.45,0.84)	0.75 (0.62,0.89)
**Non-CKD**													
N (% out of overall/CKD/non-CKD) of patients	Non-PS	NA	7,543 (15.6) vs. 32,861 (68.1)	7,829 (16.2) vs. 32,861 (68.1)	NA	7,543 (15.6) vs. 32,861 (68.1)	7,829 (16.2) vs. 32,861 (68.1)	NA	7,474 (15.5) vs. 32,826 (68.2)	7,818 (16.2) vs. 32,826 (68.2)	NA	7,474 (15.5) vs. 32,826 (68.2)	7,818 (16.2) vs. 32,826 (68.2)
	PS	4,861 (11.0) vs. 4,861 (11.0)	6,825 (15.5) vs. 6,825 (15.5)	6,729 (15.3) vs. 6,729 (15.3)	4,861 (11.0) vs. 4,861 (11.0)	6,825 (15.5) vs. 6,825 (15.5)	6,729 (15.3) vs. 6,729 (15.3)	4,821 (10.0) vs. 4,821 (10.0)	6,763 (14.1) vs. 6,763 (14.1)	6,725 (14.0) vs. 6,725 (14.0)	4,821 (10.0) vs. 4,821 (10.0)	6,763 (14.1) vs. 6,763 (14.1)	6,725 (14.0) vs. 6,725 (14.0)
Results	Non-PS	NA	0.77 (0.67,0.88)	1.02 (0.93,1.13)	NA	0.68 (0.60,0.77)	1.01 (0.93,1.09)	NA	0.56 (0.36,0.87)	0.95 (0.72,1.24)	NA	0.54 (0.36,0.80)	0.92 (0.72,1.17)
	PS	0.84 (0.68,1.02)	0.69 (0.58,0.81)	1.09 (0.97,1.22)	0.71 (0.60,0.84)	0.62 (0.54,0.70)	1.09 (0.99,1.20)	0.81 (0.44,1.50)	0.64 (0.38,1.06)	1.10 (0.78,1.56)	0.67 (0.39,1.12)	0.54 (0.35,0.84)	1.02 (0.76,1.38
**CKD**													
N (% out of overall/CKD/non-CKD) of patients	Non-PS	NA	5,583 (12.7) vs. 29,382 (66.7)	9,071 (20.6) vs. 29,382 (66.7)	NA	5,583 (12.7) vs. 29,382 (66.7)	9,071 (20.6) vs. 29,382 (66.7)	NA	5,521 (12.6) vs. 29,326 (66.8)	9,049 (20.6) vs. 29,326 (66.9)	NA	5,521 (12.6) vs. 29,326 (66.8)	9,049 (20.6) vs. 29,326 (66.9)
	PS	4,153 (8.6) vs. 4,153 (8.6)	5,041 (10.5) vs. 5,041 (10.5)	7,522 (15.6) vs. 7,522 (15.6)	4,153 (8.6) vs. 4,153 (8.6)	5,041 (10.5) vs. 5,041 (10.5)	7,522 (15.6) vs. 7,522 (15.6)	4,111 (9.4) vs. 4,111 (9.4)	4,983 (11.4) vs. 4,983 (11.4)	7,497 (17.1) vs. 7,497 (17.1)	4,111 (9.4) vs. 4,111 (9.4)	4,983 (11.4) vs. 4,983 (11.4)	7,497 (17.1) vs. 7,497 (17.1)
Results	Non-PS	NA	0.70 (0.61,0.81)	0.91 (0.84,0.99)	NA	0.70 (0.62,0.79)	0.92 (0.86,0.98)	NA	0.66 (0.44,1.00)	0.88 (0.70,1.11)	NA	0.55 (0.38,0.82)	0.88 (0.72,1.07)
	PS	0.88 (0.72,1.06)	0.68 (0.57,0.80)	0.86 (0.78,0.95)	0.81 (0.69,0.95)	0.67 (0.59,0.77)	0.90 (0.83,0.98)	1.04 (0.60,1.81)	0.76 (0.47,1.24)	0.78 (0.60,1.03)	0.86 (0.52,1.42)	0.56 (0.36,0.86)	0.80 (0.63,1.01)

Each plot shows hazard ratios and their 95% Cis estimated using Cox regression models incrementally adjusted with the following covariates: (4) Expanded case-mix+other anti-diabetes medications analyses adjusted for expanded case-mix covariates, plus insulin and metformin.

Abbrev.: CKD, chronic kidney disease; DM, diabetes mellitus; DPP4i, dipeptidyl peptidase-4 inhibitor; GLP1a, glucagon-like peptide-1 receptor agonist; PSM, propensity score match; SGLT2i, sodium-glucose cotransporter-2 inhibitor.

Supplementary Table 8. Subgroup analysis of association of SGLT2i vs. GLP1a vs. DPP4i medication use with risk of time to first infection-related hospitalization, frequency of infection-related hospitalization, time to first genitourinary infection hospitalization, and frequency of GU infection hospitalization in expanded case-mix+laboratory+other DM medication adjusted models.

	Time to first infection-related hospitalization			Frequency of infection-related hospitalization			Time to first genitourinary-infection hospitalization			Frequency of genitourinary-infection hospitalization		
	Anti-DM Medication			Anti-DM Medication			Anti-DM Medication			Anti-DM Medication		
	DPP4i	SGLT2i	GLP1a	DPP4i	SGLT2i	GLP1a	DPP4i	SGLT2i	GLP1a	DPP4i	SGLT2i	GLP1a
**Overall**												
N (% out of overall/CKD/non-CKD) of patients	62,243 (67.5)	13,126 (14.2)	16,900 (18.3)	62,243 (67.5)	13,126 (14.2)	16,900 (18.3)	62,152 (67.5)	12,995 (14.1)	16,867 (18.3)	62,152 (67.5)	12,995 (14.1)	16,867 (18.3)
	Reference	0.74 (0.67,0.81)	0.97 (0.91,1.03)	Reference	0.69 (0.64,0.75)	0.97 (0.92,1.02)	Reference	0.61 (0.46,0.83)	0.91 (0.77,1.09)	Reference	0.55 (0.41,0.72)	0.91 (0.78,1.06)
**CKD**												
N (% out of overall/CKD/non-CKD) of patients	29,382 (66.7)	5,583 (12.7)	9,071 (20.6)	29,382 (66.7)	5,583 (12.7)	9,071 (20.6)	29,326 (66.8)	5,521 (12.6)	9,049 (20.6)	29,326 (66.8)	5,521 (12.6)	9,049 (20.6)
	Reference	0.70 (0.61,0.81)	0.91 (0.84,0.99)	Reference	0.70 (0.62,0.79)	0.92 (0.86,0.98)	Reference	0.66 (0.44,1.00)	0.88 (0.70,1.11)	Reference	0.55 (0.38,0.82)	0.88 (0.72,1.07)
**Non-CKD**												
N (% out of overall/CKD/non-CKD) of patients	32,861 (68.1)	7,543 (15.6)	7,829 16.2)	32,861 (68.1)	7,543 (15.6)	7,829 16.2)	32,826 (68.2)	7,474 (15.5)	7,818 (16.2)	32,826 (68.2)	7,474 (15.5)	7,818 (16.2)
	Reference	0.77 (0.67,0.88)	1.02 (0.93,1.13)	Reference	0.68 (0.60,0.77)	1.01 (0.93,1.09)	Reference	0.56 (0.36,0.87)	0.95 (0.72,1.24)	Reference	0.54 (0.36,0.80)	0.92 (0.72,1.17)
**CKD stage 1-2**												
N (% out of overall/CKD/non-CKD) of patients	9611 (60.8)	2933 (18.6)	3260 (20.6)	9611 (60.8)	2933 (18.6)	3260 (20.6)	9,597 (60.9)	2,899 (18.4)	3,252 (20.7)	9,597 (60.9)	2,899 (18.4)	3,252 (20.7)
	Reference	0.68 (0.55,0.84)	0.97 (0.85,1.12)	Reference	0.65 (0.55,0.78)	0.98 (0.88,1.10)	Reference	0.48 (0.24,0.95)	0.88 (0.59,1.33)	Reference	0.40 (0.21,0.76)	0.75 (0.52,1.09)
**CKD stage 3**												
N (% out of overall/CKD/non-CKD) of patients	18,371 (69.9)	2,631 (10.0)	5,265 (20.0)	18,371 (69.9)	2,631 (10.0)	5,265 (20.0)	18,334 (70.0)	2,603 (9.9)	5,252 (20.1)	18,334 (70.0)	2,603 (9.9)	5,252 (20.1)
	Reference	0.76 (0.63,0.93)	0.91 (0.82,1.02)	Reference	0.77 (0.66,0.90)	0.93 (0.85,1.01)	Reference	0.82 (0.48,1.38)	0.92 (0.68,1.24)	Reference	0.68 (0.42,1.13)	1.01 (0.78,1.29)
**CKD stage 4-5**												
N (% out of overall/CKD/non-CKD) of patients	1,400 (71.2)	19 (1.0)	546 (27.8)	1,400 (71.2)	19 (1.0)	546 (27.8)	1,395 (71.2)	19 (1.0)	545 (27.8)	1,395 (71.2)	19 (1.0)	545 (27.8)
	Reference	0.59 (0.08,4.20)	0.61 (0.43,0.86)	Reference	1.35 (0.50,3.63)	0.54 (0.41,0.73)	Reference	3.79 (0.50,28.60)	0.59 (0.23,1.50)	Reference	3.46 (0.46,26.00)	0.50 (0.20,1.22)

Each plot shows hazard ratios and their 95% Cis estimated using Cox regression models incrementally adjusted with the following covariates: (4) Expanded case-mix+other anti-diabetes medications analyses adjusted for expanded case-mix covariates, plus insulin and metformin.

Abbrev.: CKD, chronic kidney disease; DM, diabetes mellitus; DPP4i, dipeptidyl peptidase-4 inhibitor; GLP1a, glucagon-like peptide-1 receptor agonist; SGLT2i, sodium-glucose cotransporter-2 inhibitor.

Supplementary Table 9. Association of SGLT2i vs. GLP1a vs. DPP4i medication use with frequency of infection-related hospitalizations in the overall cohort (Panel A; N=92,269), CKD cohort (Panel B; N=44,036), and non-CKD cohort (Panel C; N=48,233).

	Overall			CKD			Non-CKD		
	Anti-DM Medication			Anti-DM Medication			Anti-DM Medication		
**Adjustment model**	**DPP4i**	**SGLT2i**	**GLP1a**	**DPP4i**	**SGLT2i**	**GLP1a**	**DPP4i**	**SGLT2i**	**GLP1a**
N (% out of overall/CKD/non-CKD) of patients	62,243 (67.5)	13,126 (14.2)	16,900 (18.3)	29,382 (66.7)	5,583 (12.7)	9,071 (20.6)	32,861 (68.1)	7,543 (15.6)	7,829 (16.2)
Unadjusted	Reference	0.56(0.52,0.61)	0.87(0.83,0.91)	Reference	0.57(0.51,0.64)	0.85(0.80,0.90)	Reference	0.58(0.51,0.65)	0.86(0.80,0.92)
Case-mix	Reference	0.76(0.70,0.82)	1.11(1.06,1.17)	Reference	0.77(0.69,0.87)	1.06(0.99,1.13)	Reference	0.74(0.66,0.84)	1.17(1.08,1.26)
Expanded case-mix+laboratory	Reference	0.71(0.66,0.78)	1.03(0.98,1.08)	Reference	0.72(0.64,0.80)	0.96(0.89,1.02)	Reference	0.71(0.63,0.80)	1.09(1.01,1.18)
Expanded case-mix+laboratory+other DM meds	Reference	0.69(0.64,0.75)	0.97(0.92,1.02)	Reference	0.70(0.62,0.79)	0.92(0.86,0.98)	Reference	0.68(0.60,0.77)	1.01(0.93,1.09)

Each plot shows hazard ratios and their 95% CIs estimated using Cox regression models incrementally adjusted with the following covariates: (1) Unadjusted analyses; (2) Case-mix analyses adjusted for age, sex, race, ethnicity, myocardial infarction, congestive heart failure, cardiovascular disease, and Charlson Comorbidity Index; (3) Expanded case-mix analyses adjusted for case-mix covariates, plus estimated glomerular filtration rate, urine albumin-to-creatinine ratio, serum albumin, body mass index, and glycated hemoglobin; and (4) Expanded case-mix+other anti-diabetes medications analyses adjusted for expanded case-mix covariates, plus insulin and metformin.

Abbrev.: CKD, chronic kidney disease; DM, diabetes mellitus; DPP4i, dipeptidyl peptidase-4 inhibitor; GLP1a, glucagon-like peptide-1 receptor agonist; SGLT2i, sodium-glucose cotransporter-2 inhibitor.

Supplementary Table 10. Association of SGLT2i vs. GLP1a vs. DPP4i medication use with time to the first genitourinary infection hospitalization in the overall cohort (Panel A; N=92,269), CKD cohort (Panel B; N=44,036), and non-CKD cohort (Panel C; N=48,233).

	Overall			CKD			Non-CKD		
	Anti-DM Medication			Anti-DM Medication			Anti-DM Medication		
**Adjustment model**	**DPP4i**	**SGLT2i**	**GLP1a**	**DPP4i**	**SGLT2i**	**GLP1a**	**DPP4i**	**SGLT2i**	**GLP1a**
N (% out of overall/CKD/non-CKD) of patients	62,152 (67.5)	12,995 (14.1)	16,867 (18.3)	29,326 (66.8)	5,521 (12.6)	9,049 (20.6)	32,826 (68.2)	7,474 (15.5)	7,818 (16.2)
Unadjusted	Reference	0.46(0.34,0.62)	0.70(0.59,0.82)	Reference	0.49(0.33,0.74)	0.69(0.55,0.85)	Reference	0.45(0.29,0.70)	0.69(0.53,0.89)
Case-mix	Reference	0.66(0.49,0.89)	1.06(0.89,1.25)	Reference	0.72(0.48,1.07)	1.02(0.81,1.27)	Reference	0.59(0.38,0.92)	1.10(0.85,1.43)
Expanded case-mix+laboratory	Reference	0.64(0.47,0.86)	0.99(0.83,1.18)	Reference	0.68(0.45,1.02)	0.93(0.73,1.17)	Reference	0.58(0.37,0.90)	1.04(0.79,1.36)
Expanded case-mix+laboratory+other DM meds	Reference	0.62(0.46,0.83)	0.91(0.76,1.09)	Reference	0.66(0.44,1.00)	0.88(0.70,1.11)	Reference	0.56(0.36,0.87)	0.95(0.72,1.24)

Each plot shows hazard ratios and their 95% CIs estimated using Cox regression models incrementally adjusted with the following covariates: (1) Unadjusted analyses; (2) Case-mix analyses adjusted for age, sex, race, ethnicity, myocardial infarction, congestive heart failure, cardiovascular disease, and Charlson Comorbidity Index; (3) Expanded case-mix analyses adjusted for case-mix covariates, plus estimated glomerular filtration rate, urine albumin-to-creatinine ratio, serum albumin, body mass index, and glycated hemoglobin; and (4) Expanded case-mix+other anti-diabetes medications analyses adjusted for expanded case-mix covariates, plus insulin and metformin.

Abbrev.: CKD, chronic kidney disease; DM, diabetes mellitus; DPP4i, dipeptidyl peptidase-4 inhibitor; GLP1a, glucagon-like peptide 1 receptor agonist; SGLT2i, sodium-glucose cotransporter 2 inhibitor.

Supplementary Table 11. Association of SGLT2i vs. GLP1a vs. DPP4i medication use with frequency of genitourinary infection hospitalizations in the overall cohort (Panel A; N=92,269), CKD cohort (Panel B; N=44,036), and non-CKD cohort (Panel C; N=48,233).

	Overall			CKD			Non-CKD		
	Anti-DM Medication			Anti-DM Medication			Anti-DM Medication		
**Adjustment model**	**DPP4i**	**SGLT2i**	**GLP1a**	**DPP4i**	**SGLT2i**	**GLP1a**	**DPP4i**	**SGLT2i**	**GLP1a**
N (% out of overall/CKD/non-CKD) of patients	62,046 (67.5)	12,982 (14.2)	16,838 (18.3)	29,326 (66.8)	5,521 (12.6)	9,049 (20.6)	32,826 (68.2)	7,474 (15.5)	7,818 (16.2)
Unadjusted	Reference	0.42(0.32,0.55)	0.71(0.61,0.82)	Reference	0.43(0.29,0.63)	0.72(0.60,0.87)	Reference	0.42(0.28,0.63)	0.66(0.52,0.82)
Case-mix	Reference	0.60(0.46,0.79)	1.06(0.91,1.23)	Reference	0.63(0.43,0.92)	1.07(0.88,1.30)	Reference	0.57(0.39,0.86)	1.03(0.81,1.30)
Expanded case-mix+laboratory	Reference	0.57(0.43,0.75)	1.00(0.85,1.16)	Reference	0.57(0.39,0.84)	0.93(0.76,1.14)	Reference	0.56(0.38,0.84)	1.02(0.80,1.30)
Expanded case-mix+laboratory+other DM meds	Reference	0.55(0.41,0.72)	0.91(0.78,1.06)	Reference	0.55(0.38,0.82)	0.88(0.72,1.07)	Reference	0.54(0.36,0.80)	0.92(0.72,1.17)

Each plot shows hazard ratios and their 95% CIs estimated using Cox regression models incrementally adjusted with the following covariates: (1) Unadjusted analyses; (2) Case-mix analyses adjusted for age, sex, race, ethnicity, myocardial infarction, congestive heart failure, cardiovascular disease, and Charlson Comorbidity Index; (3) Expanded case-mix analyses adjusted for case-mix covariates, plus estimated glomerular filtration rate, urine albumin-to-creatinine ratio, serum albumin, body mass index, and glycated hemoglobin; and (4) Expanded case-mix+other anti-diabetes medications analyses adjusted for expanded case-mix covariates, plus insulin and metformin.
Abbrev.: CKD, chronic kidney disease; DM, diabetes mellitus; DPP4i, dipeptidyl peptidase-4 inhibitor; GLP1a, glucagon-like peptide-1 receptor agonist; SGLT2i, sodium-glucose cotransporter-2 inhibitor.

Supplementary Table 12. Association of SGLT2i vs. GLP1a vs. DPP4i medication use with risk of acute kidney injury in the overall cohort, CKD cohort, and non-CKD cohort with serum creatinine data.

	Overall			CKD			Non-CKD		
	Anti-DM Medication			Anti-DM Medication			Anti-DM Medication		
**Adjustment model**	**DPP4i**	**SGLT2i**	**GLP1a**	**DPP4i**	**SGLT2i**	**GLP1a**	**DPP4i**	**SGLT2i**	**GLP1a**
**Within 48 hours**									
N (% out of overall/CKD/non-CKD) of patients	4,158 (72.8)	647 (11.3)	910 (15.9)	2,235 (73.0)	283 (9.2)	544 (17.8)	1,923 (72.5)	364 (13.7)	366 (13.8)
Unadjusted	Reference	1.13(0.72,1.76)	1.11(0.75,1.63)	Reference	1.23(0.72,2.12)	1.10(0.72,1.70)	Reference	1.22(0.56,2.62)	0.90(0.38,2.14)
Case-mix	Reference	1.28(0.82,2.01)	1.15(0.78,1.71)	Reference	1.40(0.81,2.44)	1.12(0.72,1.75)	Reference	1.40(0.64,3.08)	1.09(0.45,2.62)
Expanded case-mix+laboratory	Reference	1.41(0.89,2.23)	1.14(0.75,1.74)	Reference	1.46(0.82,2.58)	1.18(0.73,1.90)	Reference	1.38(0.62,3.03)	1.11(0.45,2.75)
Expanded case-mix+laboratory+other DM meds	Reference	1.38(0.87,2.18)	1.09(0.71,1.66)	Reference	1.47(0.83,2.60)	1.15(0.71,1.87)	Reference	1.32(0.60,2.92)	1.01(0.40,2.51)
**Within 7 days**									
N (% out of overall/CKD/non-CKD) of patients	5,783 (70.3)	1,016 (12.3)	1,433 (17.4)	3,134 (70.1)	461 (10.3)	873 (19.5)	2,649 (70.4)	555 (14.7)	560 (14.9)
Unadjusted	Reference	1.16(0.91,1.47)	1.08(0.87,1.34)	Reference	1.06(0.77,1.44)	0.97(0.76,1.24)	Reference	1.69(1.13,2.50)	1.19(0.76,1.86)
Case-mix	Reference	1.29(1.01,1.65)	1.10(0.88,1.37)	Reference	1.16(0.85,1.60)	0.96(0.75,1.24)	Reference	1.76(1.17,2.64)	1.32(0.84,2.09)
Expanded case-mix+laboratory	Reference	1.53(1.19,1.97)	1.11(0.87,1.40)	Reference	1.35(0.98,1.87)	1.02(0.78,1.33)	Reference	1.83(1.21,2.76)	1.43(0.88,2.32)
Expanded case-mix+laboratory+other DM meds	Reference	1.49(1.16,1.92)	1.05(0.83,1.34)	Reference	1.35(0.97,1.86)	0.99(0.76,1.30)	Reference	1.75(1.16,2.64)	1.31(0.80,2.13)
**Within 30 days**									
N (% out of overall/CKD/non-CKD) of patients	12,635 (63.4)	3,159 (15.9)	4,127 (20.7)	6,870 (63.4)	1,447 (13.3)	2,523 (23.3)	5,765 (63.5)	1,712 (18.9)	1,604 (17.7)
Unadjusted	Reference	1.45(1.30,1.61)	1.11(0.9961941,1.23)	Reference	1.37(1.20,1.57)	0.98(0.87,1.11)	Reference	1.99(1.66,2.39)	1.28(1.03,1.59)
Case-mix	Reference	1.72(1.54,1.92)	1.18(1.06,1.32)	Reference	1.58(1.38,1.81)	1.02(0.90,1.16)	Reference	2.22(1.85,2.67)	1.41(1.13,1.75)

Expanded case-mix+laboratory	Reference	1.99(1.78,2.22)	1.15(1.02,1.29)	Reference	1.88(1.63,2.16)	1.06(0.93,1.21)	Reference	2.25(1.87,2.72)	1.45(1.15,1.82)
Expanded case-mix+laboratory+other DM meds	Reference	1.93(1.73,2.16)	1.10(0.98,1.23)	Reference	1.84(1.60,2.13)	1.03(0.90,1.18)	Reference	2.16(1.78,2.61)	1.34(1.06,1.69)
**Within 60 days**									
N (% out of overall/CKD/non-CKD) of patients	21,130 (62.6)	5,370 (15.9)	7,263 (21.5)	11,001 (62.3)	2,377 (13.5)	4,273 (24.2)	10,129 (62.9)	2,993 (18.6)	2,990 (18.6)
Unadjusted	Reference	1.35(1.24,1.46)	1.07(0.99,1.15)	Reference	1.28(1.15,1.42)	0.96(0.87,1.05)	Reference	1.74(1.52,1.98)	1.17(1.01,1.36)
Case-mix	Reference	1.58(1.45,1.71)	1.14(1.05,1.23)	Reference	1.46(1.31,1.63)	0.99(0.91,1.09)	Reference	1.92(1.68,2.20)	1.28(1.09,1.49)
Expanded case-mix+laboratory	Reference	1.79(1.64,1.94)	1.07(0.98,1.17)	Reference	1.72(1.54,1.92)	1.01(0.91,1.11)	Reference	1.91(1.66,2.19)	1.25(1.06,1.47)
Expanded case-mix+laboratory+other DM meds	Reference	1.74(1.60,1.89)	1.03(0.94,1.12)	Reference	1.69(1.51,1.89)	0.98(0.88,1.08)	Reference	1.84(1.60,2.11)	1.17(1.00,1.38)
**Within 90 days**									
N (% out of overall/CKD/non-CKD) of patients	30,773 (63.9)	7,276 (15.1)	10,139 (21.0)	15,471 (63.4)	3,181 (13.0)	5,762 (23.6)	15,302 (64.4)	4,095 (17.2)	4,377 (18.4)
Unadjusted	Reference	1.36(1.27,1.46)	1.12(1.04,1.19)	Reference	1.28(1.17,1.40)	1.02(0.95,1.10)	Reference	1.76(1.57,1.97)	1.16(1.02,1.31)
Case-mix	Reference	1.57(1.47,1.69)	1.18(1.11,1.27)	Reference	1.45(1.33,1.59)	1.05(0.97,1.13)	Reference	1.91(1.71,2.14)	1.26(1.11,1.43)
Expanded case-mix+laboratory	Reference	1.74(1.62,1.87)	1.09(1.01,1.17)	Reference	1.67(1.52,1.84)	1.03(0.95,1.12)	Reference	1.89(1.69,2.12)	1.23(1.07,1.40)
Expanded case-mix+laboratory+other DM meds	Reference	1.69(1.58,1.82)	1.03(0.96,1.11)	Reference	1.63(1.48,1.79)	0.99(0.91,1.07)	Reference	1.83(1.63,2.05)	1.16(1.01,1.32)

Each plot shows hazard ratios and their 95% CIs estimated using Cox regression models incrementally adjusted with the following covariates: (1) Unadjusted analyses; (2) Case-mix analyses adjusted for age, sex, race, ethnicity, myocardial infarction, congestive heart failure, cardiovascular disease, and Charlson Comorbidity Index; (3) Expanded case-mix analyses adjusted for case-mix covariates, plus estimated glomerular filtration rate, urine albumin-to-creatinine ratio, serum albumin, body mass index, and glycated hemoglobin; and (4) Expanded case-mix+other anti-diabetes medications analyses adjusted for expanded case-mix covariates, plus insulin and metformin.

Abbrev.: CKD, chronic kidney disease; DM, diabetes mellitus; DPP4i, dipeptidyl peptidase-4 inhibitor; GLP1a, glucagon-like peptide-1 receptor agonist; SGLT2i, sodium-glucose cotransporter-2 inhibitor.

Supplementary Table 13. Comparison of results from propensity score (PS) matched vs. non-PS matched analyses incorporating expanded case-mix+laboratory+other DM medication covariates examining associations of SGLT2i vs. GLP1a vs. DPP4i medication use with risk of time to first acute kidney injury, amputation, and diabetic ketoacidosis.

		**Time to first AKI within 48-hours**			**Time to first amputation**			**Time to first DKA**		
		**SGLT2i vs. GLP1a**	**SGLT2i vs. DPP4i**	**GLP1a vs. DPP4i**	**SGLT2i vs. GLP1a**	**SGLT2i vs. DPP4i**	**GLP1a vs. DPP4i**	**SGLT2i vs. GLP1a**	**SGLT2i vs. DPP4i**	**GLP1a vs. DPP4i**
**Overall**										
N (% out of overall/CKD/non-CKD) of patients	Non-PS	NA	647 (11.3) vs. 4,158 (72.8)	910(15.9) vs. 4,158 (72.8)	NA	11,601 (13.1) vs. 60,619 (68.7)	16,061 (18.2) vs. 60,619 (68.7)	NA	12,885 (14.1) vs. 62,026 (67.7)	16,773 (18.3) vs. 62,026 (67.7)
	PS	464 (8.1) vs. 464 (8.1)	593 (10.4) vs. 593 (10.4)	833 (14.6) vs. 833 (14.6)	8,233 (9.3) vs. 8,233 (9.3)	10,482 (11.9) vs. 10,482 (11.9)	13,587 (15.4) vs. 13,587 (15.4)	8,964 (9.8) vs. 8,964 (9.8)	11,643 (12.7) vs. 11,643 (12.7)	14,190 (15.5) vs. 14,190 (15.5)
Results	Non-PS	NA	1.38(0.87,2.18)	1.09(0.71,1.66)	NA	0.94(0.67,1.31)	1.19(0.98,1.45)	NA	1.88(1.52,2.33)	1.77(1.50,2.09)
	PS	1.30(0.65,2.61)	1.35(0.69,2.63)	1.15(0.68,1.95)	0.73(0.45,1.19)	0.75(0.49,1.13)	1.05(0.83,1.33)	1.28(0.95,1.74)	1.57(1.19,2.08)	1.38(1.13,1.69)
**Non-CKD**										
N (% out of overall/CKD/non-CKD) of patients	Non-PS	NA	364 (13.7) vs. 1,923 (72.5)	366 (13.8) vs. 1,923 (72.5)	NA	6,764 (14.6) vs. 32,082 (69.2)	7,518 (16.2) vs. 32,082 (69.2)	NA	7,422 (15.5) vs. 32,772 (68.3)	7,793 (16.2) vs. 32,772 (68.3)
	PS	228 (8.6) vs. 228 (8.6)	332 (12.5) vs. 332 (12.5)	336 (12.7) vs. 336 (12.7)	4,448 (9.6) vs. 4,448 (9.6)	6,117 (13.2) vs. 6,117 (13.2)	6,460 (13.9) vs. 6,460 (13.9)	4,796 (10.0) vs. 4,796 (10.0)	6,716 (14.0) vs. 6,716 (14.0)	6,704 (14.0) vs. 6,704 (14.0)
Results	Non-PS	NA	1.32(0.60,2.92)	1.01(0.40,2.51)	NA	1.13(0.70,1.82)	1.52(1.12,2.07)	NA	1.54(1.12,2.11)	1.80(1.42,2.28)
	PS	1.67(0.40,6.98)	1.41(0.45,4.45)	0.66(0.24,1.87)	0.77(0.40,1.46)	0.78(0.43,1.41)	1.48(1.01,2.19)	1.10(0.72,1.68)	1.29(0.87,1.93)	1.32(0.99,1.77)
**CKD**										
N (% out of overall/CKD/non-CKD) of patients	Non-PS	NA	283 (9.2) vs. 2,235 (73.0)	544 (17.8) vs. 2,235 (73.0)	NA	4,837 (11.5) vs. 28,537 (68.1)	8,543 (20.4) vs. 28,537 (68.1)	NA	5,463 (12.5) vs. 29,254 (66.9)	8,980 (20.6) vs. 29,254 (66.9)
	PS	217 (7.1) vs. 217 (7.1)	260 (8.5) vs. 260 (8.5)	481 (15.7) vs. 481 (15.7)	3,694 8.8) vs. 3,694 (8.8)	4,365 (10.4) vs. 4,365 (10.4)	7,110 (17.0) vs. 7,110 (17.0)	4,073 (9.3) vs. 4,073 (9.3)	4,928 (11.3) vs. 4,928 (11.3)	7,454 (17.1) vs. 7,454 (17.1)
Results	Non-PS	NA	1.47(0.83,2.60)	1.15(0.71,1.87)	NA	0.77(0.48,1.23)	0.94(0.73,1.22)	NA	2.14(1.59,2.87)	1.54(1.22,1.94)
	PS	1.39(0.56,3.46)	1.66(0.69,4.00)	1.00(0.56,1.81)	0.50(0.25,0.98)	0.73(0.41,1.30)	1.04(0.77,1.40)	1.33(1.004,1.76)	1.94(1.31,2.86)	1.43(0.95,2.15)

Each plot shows hazard ratios and their 95% CIs estimated using Cox regression models incrementally adjusted with the following covariates: (4) Expanded case-mix+other anti-diabetes medications analyses adjusted for expanded case-mix covariates, plus insulin and metformin.
Abbrev.: AKI, acute kidney injury; CKD, chronic kidney disease; DKA, diabetic ketoacidosis; DPP4i, dipeptidyl peptidase-4 inhibitor; GLP1a, glucagon-like peptide-1 receptor agonist; SGLT2i, sodium-glucose cotransporter-2 inhibitor.

Supplementary Table 14. Subgroup analysis of association of SGLT2i vs. GLP1a vs. DPP4i medication use with risk of time to first acute kidney injury, amputation, and diabetic ketoacidosis in expanded case-mix+laboratory+other DM medication adjusted models.

	Time to first AKI			Time to first amputation			Time to first DKA		
	Anti-DM Medication			Anti-DM Medication			Anti-DM Medication		
	DPP4i	SGLT2i	GLP1a	DPP4i	SGLT2i	GLP1a	DPP4i	SGLT2i	GLP1a
**Overall**									
N (% out of overall/CKD/non-CKD) of patients	4,158 (72.8)	647 (11.3)	910 (15.9)	60,619 (68.7)	11,601 (13.1)	16,061 (18.2)	62,026 (67.7)	12,885 (14.2)	16,773 (18.3)
	Reference	1.38(0.87,2.18)	1.09(0.71,1.66)	Reference	0.94(0.67,1.31)	1.19(0.98,1.45)	Reference	1.88(1.52,2.33)	1.77(1.50,2.09)
**CKD**									
N (% out of overall/CKD/non-CKD) of patients	2,235 (73.0)	283 (9.2)	544 (17.8)	28,537 (68.1)	4,837 (11.5)	8,543 (20.4)	29,254 (66.9)	5,463 (12.5)	8,980 (20.6)
	Reference	1.47(0.83,2.60)	1.15(0.71,1.87)	Reference	0.77(0.48,1.23)	0.94(0.73,1.22)	Reference	2.14(1.59,2.87)	1.54(1.22,1.94)
**Non-CKD**									
N (% out of overall/CKD/non-CKD) of patients	1,923 (72.5)	364 (13.7)	366 (13.8)	32,082 (69.2)	6,764 (14.6)	7,518 (16.2)	32,772 (68.3)	7,422 (15.5)	7,793 (16.2)
	Reference	1.32(0.60,2.92)	1.01(0.40,2.51)	Reference	1.13(0.70,1.82)	1.52(1.12,2.07)	Reference	1.54(1.12,2.11)	1.80(1.42,2.28)
**CKD stage 1-2**									
N (% out of overall/CKD/non-CKD) of patients	645 (69.7)	139 (15.0)	141 (15.2)	9,345 (62.1)	2,602 (17.3)	3,112 (20.7)	9,574 (61.1)	2,874 (18.3)	3,232 (20.6)
	Reference	2.14(0.87,5.24)	2.23(0.85,5.87)	Reference	0.76(0.38,1.51)	1.27(0.86,1.89)	Reference	2.59(1.68,3.99)	2.04(1.41,2.94)
**CKD stage 3**									
N (% out of overall/CKD/non-CKD) of patients	1,347 (74.0)	141 (7.7)	333 (18.3)	17,841 (71.3)	2,224 (8.9)	4,943 (19.8)	18,294 (70.2)	2,572 (9.9)	5,209 (20.)
	Reference	1.67(0.77,3.62)	1.01(0.52,1.96)	Reference	0.87(0.46,1.66)	0.87(0.61,1.23)	Reference	2.11(1.39,3.20)	1.27(0.92,1.75)
**CKD stage 4-5**									
N (% out of overall/CKD/non-CKD) of patients	243 (76.9)	3 (0.9)	70 (22.2)	1,351 (73.0)	11 (0.6)	488 (26.4)	1,386 (71.4)	17 (0.9)	539 (27.8)
	Reference	NA	0.77(0.23,2.54)	Reference		0.12(0.02,0.95)	Reference		1.28(0.39,4.14)

Each plot shows hazard ratios and their 95% CIs estimated using Cox regression models incrementally adjusted with the following covariates: (4) Expanded case-mix+other anti-diabetes medications analyses adjusted for expanded case-mix covariates, plus insulin and metformin.
Abbrev.: AKI, acute kidney injury; CKD, chronic kidney disease; DM, diabetes mellitus; DKA, diabetic ketoacidosis; DPP4i, dipeptidyl peptidase-4 inhibitor; GLP1a, glucagon-like peptide-1 receptor agonist; SGLT2i, sodium-glucose cotransporter-2 inhibitor.

Supplementary Table 15. Drug discontinuation according to presence vs. absence of acute kidney injury (AKI) within 48-hours of initial drug prescription among patients with serum creatinine data values (N=5,715).

N (%) of Drug discontinuation	Overall	CKD	Non-CKD
Overall	647 (11)	368 (12)	279 (11)
AKI	31 (17)	26 (19)	5 (10)
Non-AKI	651 (12)	363 (12)	288 (11)

Abbrev.: AKI, acute kidney injury; CKD, chronic kidney disease; DPP4i, dipeptidyl peptidase-4 inhibitor; GLP1a, glucagon-like peptide 1 receptor agonist; SGLT2i, sodium-glucose cotransporter 2 inhibitor.

Supplementary Table 16. Association of SGLT2i vs. GLP1a vs. DPP4i medication use with risk of lower extremity amputation in the overall cohort (Panel A; N=88,281), CKD cohort (Panel B; N=41,917), non-CKD cohort (Panel C; N=46,364).

	**Overall**			**CKD**			**Non-CKD**		
	**Anti-DM Medication**			**Anti-DM Medication**			**Anti-DM Medication**		
**Adjustment model**	**DPP4i**	**SGLT2i**	**GLP1a**	**DPP4i**	**SGLT2i**	**GLP1a**	**DPP4i**	**SGLT2i**	**GLP1a**
N (% out of overall/CKD/non-CKD) of patients	60,619 (68.7)	11,601 (13.1)	16,061 (18.2)	28,537 (68.1)	4,837 (11.5)	8,543 (20.4)	32,082 (69.2)	6,764 (14.6)	7,518 (16.2)
Unadjusted	Reference	0.81(0.58,1.13)	1.18(0.99,1.42)	Reference	0.73(0.46,1.16)	1.05(0.83,1.33)	Reference	1.02(0.64,1.64)	1.37(1.04,1.82)
Case-mix	Reference	0.93(0.67,1.30)	1.21(1.00,1.45)	Reference	0.80(0.50,1.28)	0.97(0.76,1.24)	Reference	1.14(0.71,1.84)	1.53(1.14,2.05)
Expanded case-mix+laboratory	Reference	0.98(0.70,1.37)	1.29(1.06,1.57)	Reference	0.79(0.49,1.26)	0.99(0.77,1.28)	Reference	1.19(0.74,1.92)	1.68(1.24,2.28)
Expanded case-mix+laboratory+other DM meds	Reference	0.94(0.67,1.31)	1.19(0.98,1.45)	Reference	0.77(0.48,1.23)	0.94(0.73,1.22)	Reference	1.13(0.70,1.82)	1.52(1.12,2.07)

Each plot shows hazard ratios and their 95% CIs estimated using Cox regression models incrementally adjusted with the following covariates: (1) Unadjusted analyses; (2) Case-mix analyses adjusted for age, sex, race, ethnicity, myocardial infarction, congestive heart failure, cardiovascular disease, and Charlson Comorbidity Index; (3) Expanded case-mix analyses adjusted for case-mix covariates, plus estimated glomerular filtration rate, urine albumin-to-creatinine ratio, serum albumin, body mass index, and glycated hemoglobin; and (4) Expanded case-mix+other anti-diabetes medications analyses adjusted for expanded case-mix covariates, plus insulin and metformin.
Abbrev.: CKD, chronic kidney disease; DM, diabetes mellitus; DPP4i, dipeptidyl peptidase-4 inhibitor; GLP1a, glucagon-like peptide-1 receptor agonist; SGLT2i, sodium-glucose cotransporter-2 inhibitor.

Supplementary Table 17. Association of SGLT2i vs. GLP1a vs. DPP4i medication use with risk of diabetic ketoacidosis in the overall cohort (Panel A; N=91,684), CKD cohort (Panel B; N=43,697), and non-CKD cohort (Panel C; N=47,987).

	Overall			CKD			Non-CKD		
	Anti-DM Medication			Anti-DM Medication			Anti-DM Medication		
**Adjustment model**	**DPP4i**	**SGLT2i**	**GLP1a**	**DPP4i**	**SGLT2i**	**GLP1a**	**DPP4i**	**SGLT2i**	**GLP1a**
N (% out of overall/CKD/non-CKD) of patients	62,026 (67.7)	12,885 (14.1)	16,773 (18.3)	29,254 (66.9)	5,463 (12.5)	8,980 (20.6)	32,772 (68.3)	7,422 (15.5)	7,793 (16.2)
Unadjusted	Reference	1.54(1.25,1.91)	1.53(1.31,1.78)	Reference	1.87(1.40,2.50)	1.47(1.19,1.81)	Reference	1.31(0.96,1.79)	1.59(1.28,1.98)
Case-mix	Reference	1.81(1.46,2.24)	1.51(1.29,1.77)	Reference	2.16(1.61,2.89)	1.43(1.15,1.78)	Reference	1.49(1.09,2.04)	1.58(1.26,1.99)
Expanded case-mix+laboratory	Reference	2.00(1.62,2.48)	2.02(1.71,2.38)	Reference	2.22(1.65,2.98)	1.66(1.31,2.09)	Reference	1.65(1.20,2.26)	2.08(1.64,2.64)
Expanded case-mix+laboratory+other DM meds	Reference	1.88(1.52,2.33)	1.77(1.50,2.09)	Reference	2.14(1.59,2.87)	1.54(1.22,1.94)	Reference	1.54(1.12,2.11)	1.80(1.42,2.28)

Each plot shows hazard ratios and their 95% CIs estimated using Cox regression models incrementally adjusted with the following covariates: (1) Unadjusted analyses; (2) Case-mix analyses adjusted for age, sex, race, ethnicity, myocardial infarction, congestive heart failure, cardiovascular disease, and Charlson Comorbidity Index; (3) Expanded case-mix analyses adjusted for case-mix covariates, plus estimated glomerular filtration rate, urine albumin-to-creatinine ratio, serum albumin, body mass index, and glycated hemoglobin; and (4) Expanded case-mix+other anti-diabetes medications analyses adjusted for expanded case-mix covariates, plus insulin and metformin.
Abbrev.: CKD, chronic kidney disease; DM, diabetes mellitus; DPP4i, dipeptidyl peptidase-4 inhibitor; GLP1a, glucagon-like peptide-1 receptor agonist; SGLT2i, sodium-glucose cotransporter-2 inhibitor.

Supplementary Figure 1. Study cohort creation for infection-related hospitalization analyses.

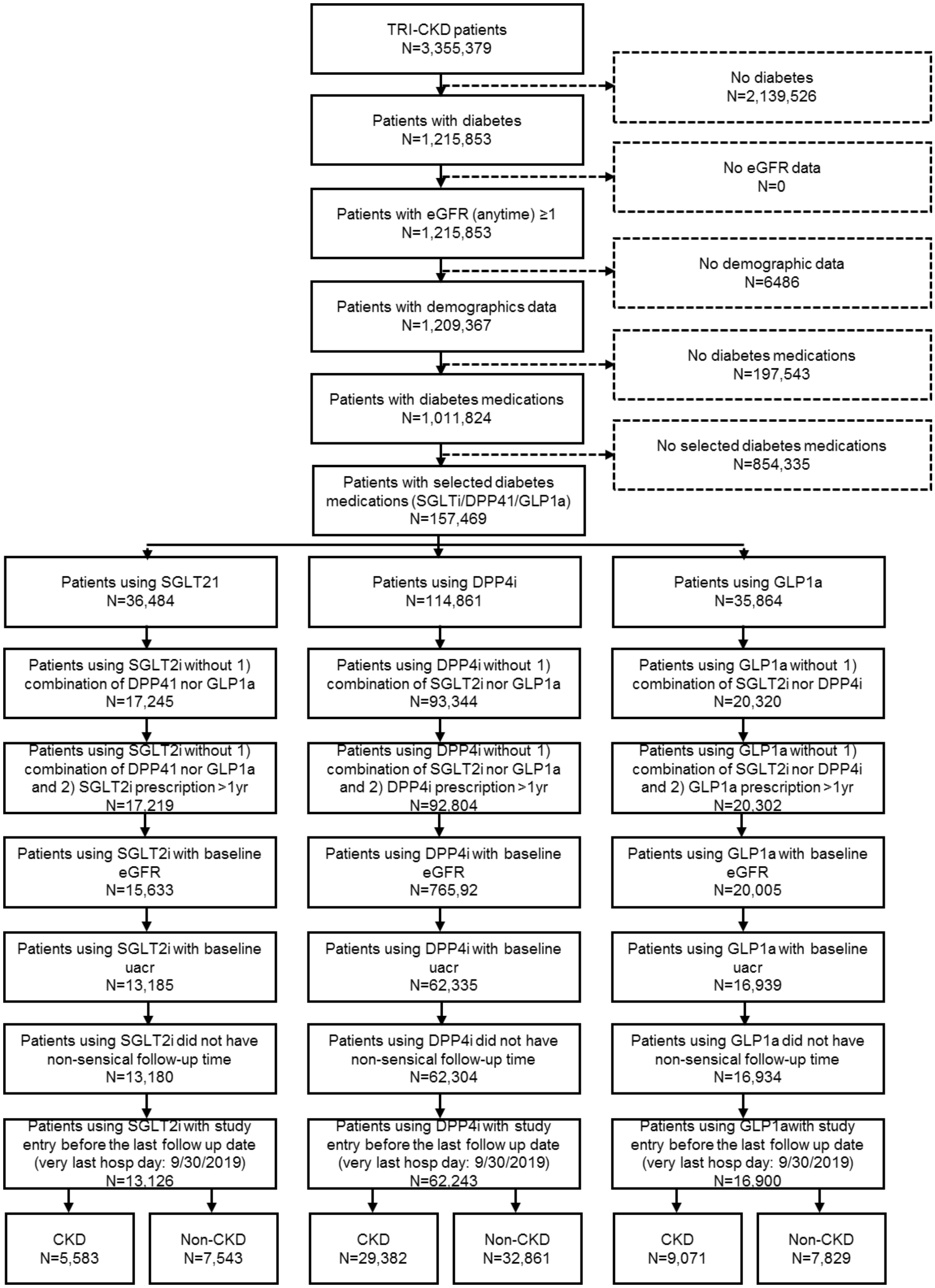

Supplementary Figure 2. Study cohort creation for acute kidney injury analyses.

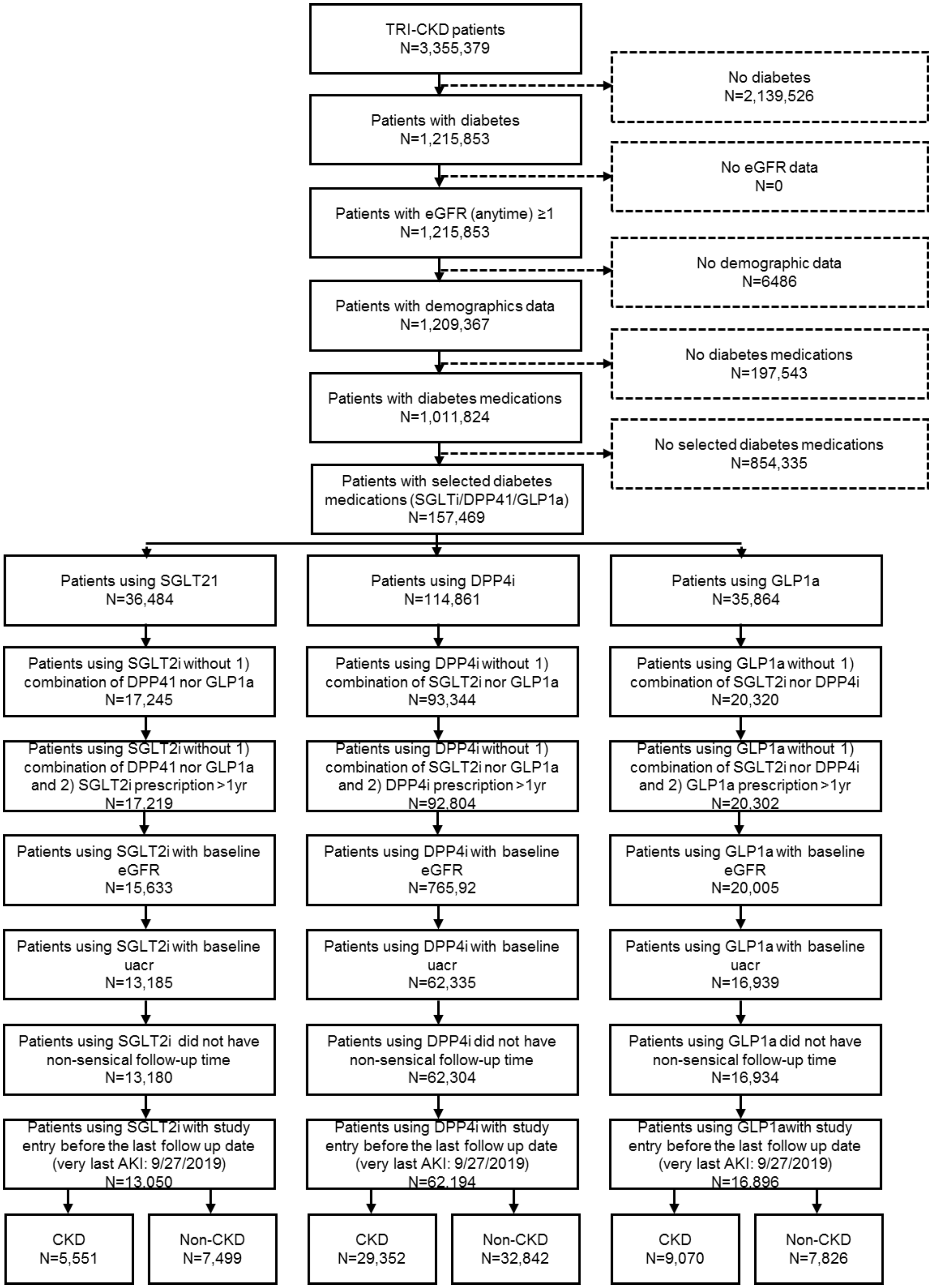

Supplementary Figure 3. Study cohort creation for amputation analyses.

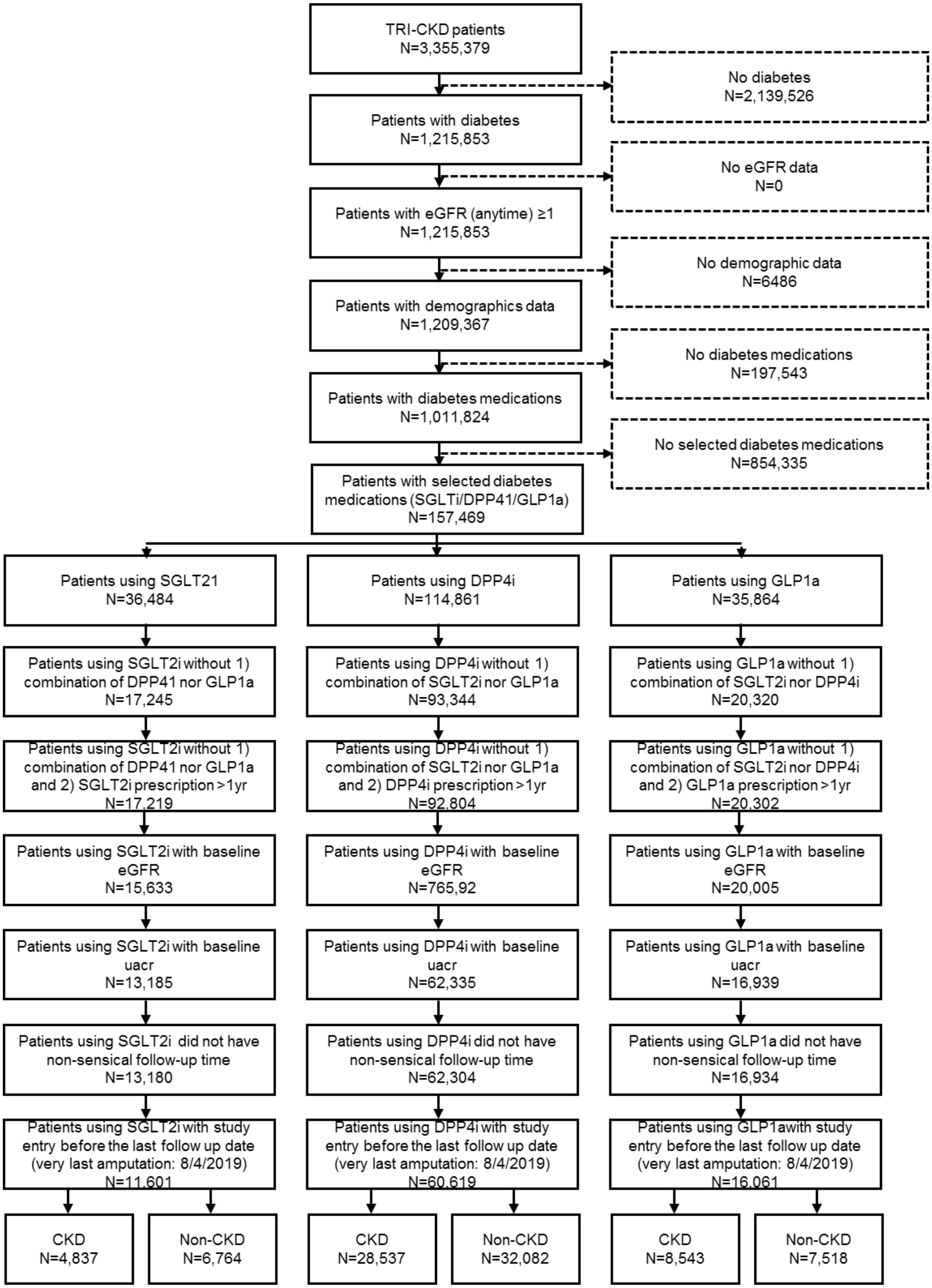

Supplementary Figure 4. Study cohort creation for diabetic ketoacidosis analyses.

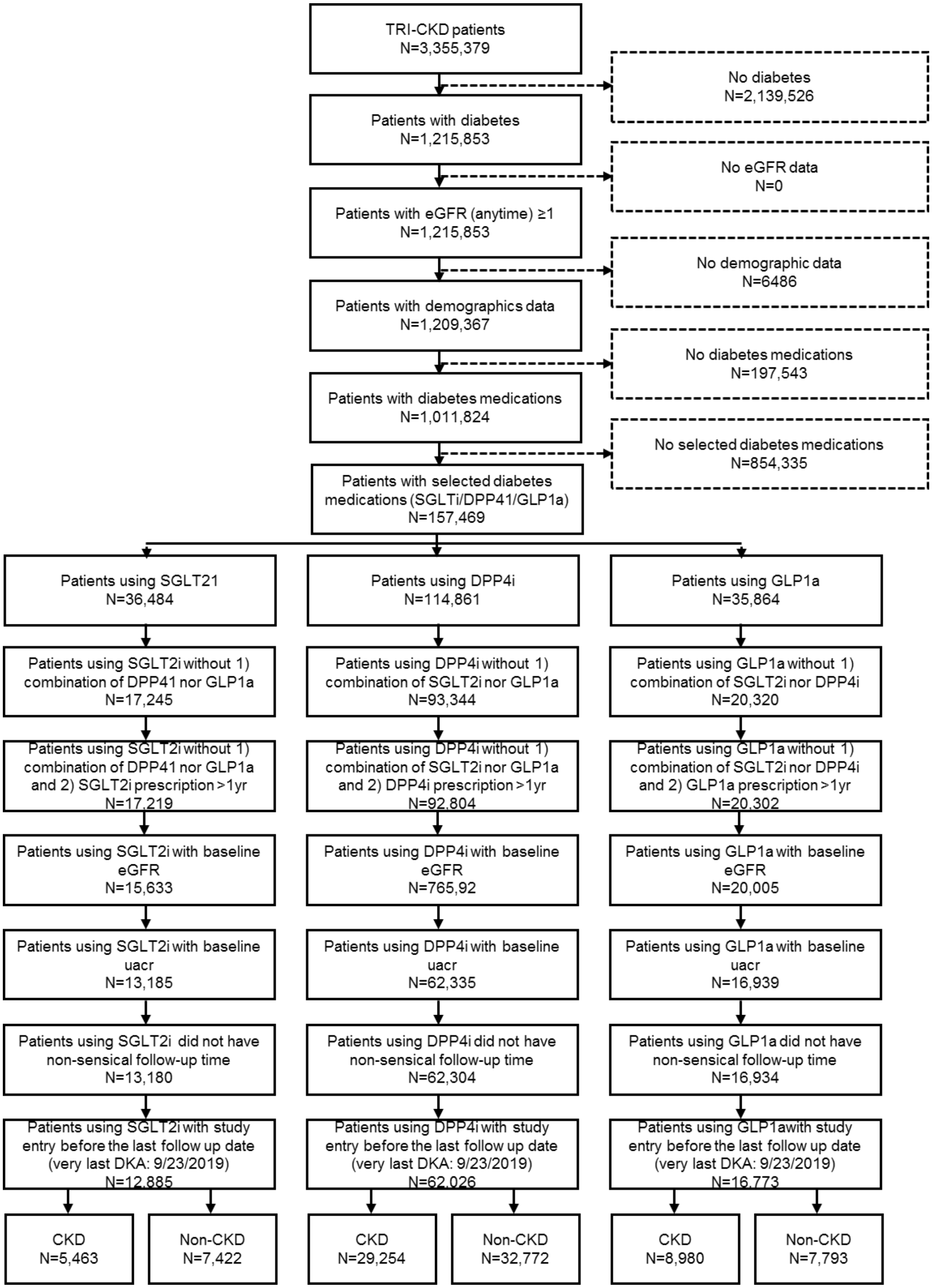

Supplementary Figure 5. Association of SGLT2i vs. GLP1a vs. DPP4i medication use with time to the first genitourinary infection hospitalization in the overall cohort (Panel A; N=92,269), CKD cohort (Panel B; N=44,036), and non-CKD cohort (Panel C; N=48,233).

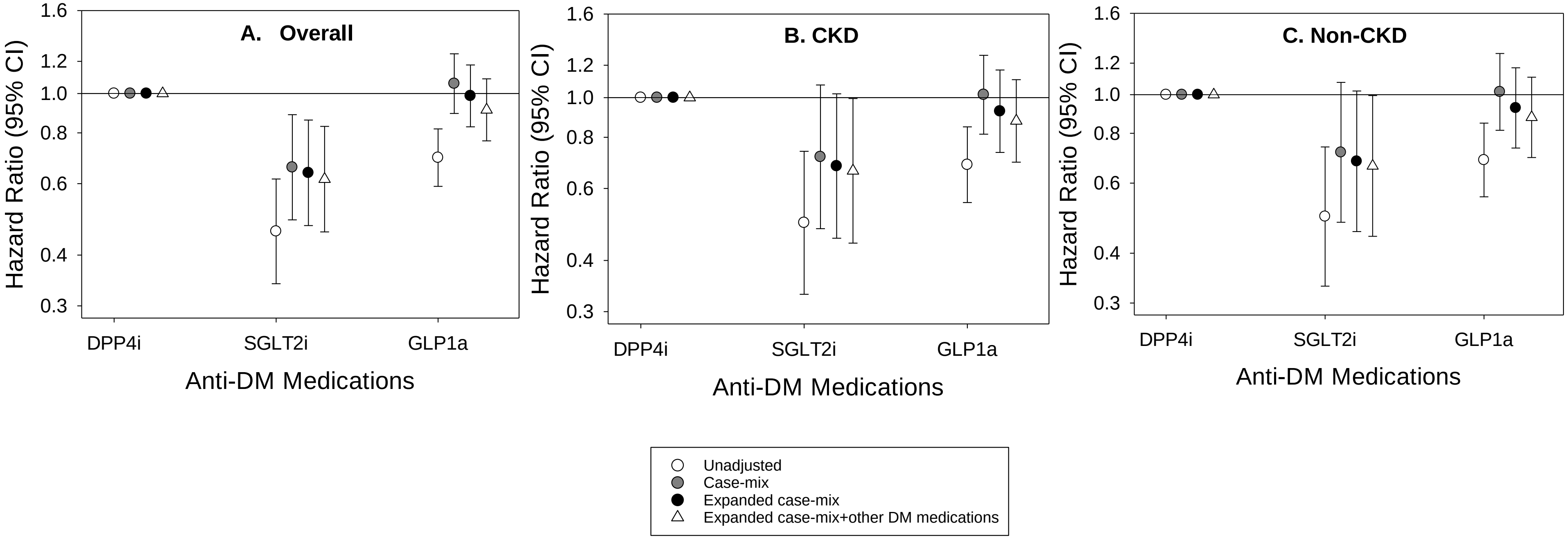

Supplementary Figure 6. Association of SGLT2i vs. GLP1a vs. DPP4i medication use with frequency of genitourinary infection hospitalizations in the overall cohort (Panel A; N=92,269), CKD cohort (Panel B; N=44,036), and non-CKD cohort (Panel C; N=48,233).

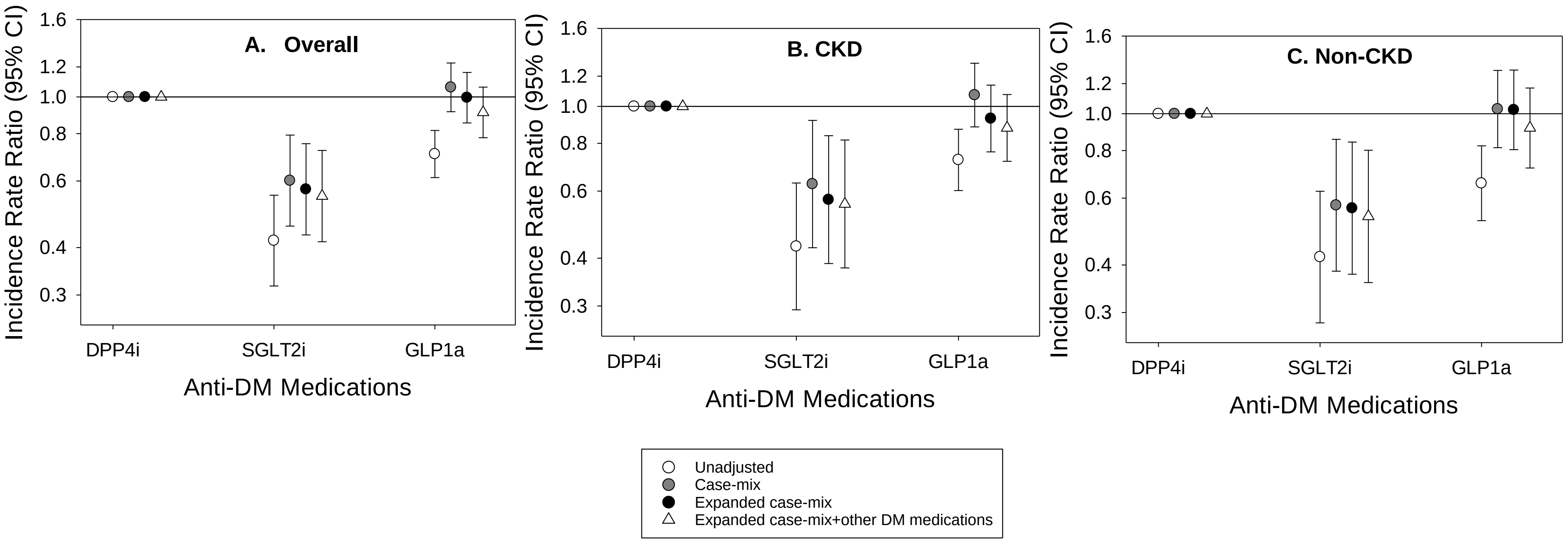

Supplement: Supplementary Figs. S1–S6 and Tables S1–S17 [file mmc1.pdf]
