# Supplementary material for: Safety of SGLT2 inhibitors, DPP-4 inhibitors, and GLP-1 receptor agonists in US veterans with and without chronic kidney disease: a population-based study
Source: Lancet Reg Health Am. 2024 Jun 18;36:100814. doi: 10.1016/j.lana.2024.100814 (PMC11237921; doi:10.1016/j.lana.2024.100814)
Supplement: AntiDMMed_Safety_LanReg_CL [file mmc2.docx]

| UNIVERSITY OF CALIFORNIA LOS ANGELES  **BERKELEY _η_ DAVIS _η_ IRVINE _η_ LOS ANGELES _η_ RIVERSIDE _η_ SAN DIEGO _η_ SAN FRANCISCO** | |  | UCLA  **SANTA BARBARA _η_ SANTA CRUZ** |
| --- | --- | --- | --- |
| April 25^th^, 2024  Dr. Taissa Vila (Editor-in-Chief)  Dr. Orison Woolcott (Senior Editor)  The Lancet Regional Health – Americas |  | | |

Re: Revised manuscript submission titled, **“Safety of SGLT2 Inhibitors, DPP-4 Inhibitors, and GLP-1 Agonists in US Veterans with and without Chronic Kidney Disease: A Population Health Study”** (Manuscript#: TLRHAMERICAS-D-23-00519R1)

Dear Dr. Vila and Dr. Woolcott,

Thank you for your recent review of our above-mentioned manuscript and for the opportunity to submit a revised version for consideration of publication in *The Lancet Regional Health – Americas*. We thank you and the reviewers for your time, consideration, and valuable comments in regards to the original and most recently revised manuscripts. We have addressed the points raised during the editorial process and feel that the manuscript is greatly enhanced as a result. Elsewhere in this submission is a detailed response to the points raised in the editorial process.

We thank you in advance for your consideration of this manuscript and look forward to your response.

Sincerely,

Connie M. Rhee, MD, MSc
Chief, Division of Nephrology, Departments of Medicine

VA Greater Los Angeles Health System

Professor of Medicine, David Geffen School of Medicine

University of California Los Angeles

11301 Wilshire Blvd, Los Angeles, California 90073

Tel: 310-478-3711

Email: [crhee@mednet.ucla.edu](mailto:crhee@mednet.ucla.edu)
